# The potent analgesia of intrathecal 2R, 6R-HNK via TRPA1 inhibition in LF-PENS-induced chronic primary pain model

**DOI:** 10.1186/s10194-023-01667-1

**Published:** 2023-10-19

**Authors:** An-Ran Liu, Zhen-Jia Lin, Ming Wei, Yuan Tang, Hui Zhang, Xiang-Ge Peng, Ying Li, Yu-Fan Zheng, Zhi Tan, Li-Jun Zhou, Xia Feng

**Affiliations:** 1 Department of Anesthesiology and Pain Clinic, First Affiliated Hospital of Sun Yat-Sen University, No.58, 2Nd Zhongshan Road, Yuexiu District, Guangzhou, 510080 China; 2 Department of Physiology and Pain Research Center, Zhongshan School of Medicine, Guangdong Province Key Laboratory of Brain Function and Disease, Sun Yat-Sen University, No.74, 2Nd Zhongshan Road, Yuexiu District, Guangzhou, 510080 China; 3 Department of Anesthesiology, Guangdong Second Provincial General Hospital, No.466, Mid Xingang Road, Haizhu District, Guangzhou, 510317 China

**Keywords:** Chronic primary pain, 2R, 6R-HNK, DRG, CGRP, TRPA1

## Abstract

**Background:**

Chronic primary pain (CPP) is an intractable pain of unknown cause with significant emotional distress and/or dysfunction that is a leading factor of disability globally. The lack of a suitable animal model that mimic CPP in humans has frustrated efforts to curb disease progression. 2R, 6R-hydroxynorketamine (2R, 6R-HNK) is the major antidepressant metabolite of ketamine and also exerts antinociceptive action. However, the analgesic mechanism and whether it is effective for CPP are still unknown.

**Methods:**

Based on nociplastic pain is evoked by long-term potentiation (LTP)-inducible high- or low-frequency electrical stimulation (HFS/LFS), we wanted to develop a novel CPP mouse model with mood and cognitive comorbidities by noninvasive low-frequency percutaneous electrical nerve stimulation (LF-PENS). Single/repeated 2R, 6R-HNK or other drug was intraperitoneally (i.p.) or intrathecally (i.t.) injected into naïve or CPP mice to investigate their analgesic effect in CPP model. A variety of behavioral tests were used to detect the changes in pain, mood and memory. Immunofluorescent staining, western blot, reverse transcription-quantitative real-time polymerase chain reaction (RT-qPCR) and calcium imaging of in cultured dorsal root ganglia (DRG) neurons by Fluo-8-AM were used to elucidate the role and mechanisms of 2R, 6R-HNK in vivo or in vitro.

**Results:**

Intrathecal 2R, 6R-HNK, rather than intraperitoneal 2R, 6R-HNK or intrathecal S-Ketamine, successfully mitigated HFS-induced pain. Importantly, intrathecal 2R, 6R-HNK displayed effective relief of bilateral pain hypersensitivity and depressive and cognitive comorbidities in a dose-dependent manner in LF-PENS-induced CPP model. Mechanically, 2R, 6R-HNK markedly attenuated neuronal hyperexcitability and the upregulation of calcitonin gene-related peptide (CGRP), transient receptor potential ankyrin 1 (TRPA1) or vanilloid-1 (TRPV1), and vesicular glutamate transporter-2 (VGLUT2) in peripheral nociceptive pathway. In addition, 2R, 6R-HNK suppressed calcium responses and CGRP overexpression in cultured DRG neurons elicited by the agonists of TRPA1 or/and TRPV1. Strikingly, the inhibitory effects of 2R, 6R-HNK on these pain-related molecules and mechanical allodynia were substantially occluded by TRPA1 antagonist menthol.

**Conclusions:**

In the newly designed CPP model, our findings highlighted the potential utility of intrathecal 2R, 6R-HNK for preventing and therapeutic modality of CPP. TRPA1-mediated uprgulation of CGRP and neuronal hyperexcitability in nociceptive pathways may undertake both unique characteristics and solving process of CPP.

**Supplementary Information:**

The online version contains supplementary material available at 10.1186/s10194-023-01667-1.

## Highlights

1) We first establish the CPP mouse model by LF-PENS to mimic nociplastic pain.

2) Intrathecal 2R, 6R-HNK effectively alleviates LF-PENS-induced CPP in mouse models by inhibiting TRPA1 in DRG neurons.

**Supplementary Information:**

The online version contains supplementary material available at 10.1186/s10194-023-01667-1.

## Background

Chronic primary pain is the first subcategory of chronic pain that cannot be unaccounted by chronic secondary conditions and is characterized by severe functional interference or emotional distress [1]. The diagnostic code for CPP in the 11th revision of the International Statistical Classification of Diseases and Related Health Problems (ICD-11) is MG30.0, which includes chronic widespread pain (such as fibromyalgia), complex regional pain syndrome (CRPS), chronic primary headache or orofacial pain [such as chronic migraine, trigeminal autonomic pain, chronic burning mouth syndrome (BMS)], chronic primary visceral pain [e.g. irritable bowel syndrome (IBS), chronic pelvic pain], and chronic primary musculoskeletal pain [e.g. nonspecific low back pain [2]. It is a global public health problem, and some types are relatively common and rank high for the health metric of estimated years lived with disability. However, the lack of a clear underlying etiology and suitable noninvasive animal models have hindered the exploration of the pathogenesis of CPP and the development of prevention and treatment interventions.

Nociplastic pain is functional pain defined as altered nociception without evidence of tissue or somatosensory damage [3]. Mechanistically distinct from nociceptive and neuropathic pain, it is more suitable to underlie CPP conditions [4]. The IASP clinical criteria and grading system for nociplastic pain replaced the 2014 clinical criteria for predominant central sensitization pain affecting the musculoskeletal system [5], suggesting that nociplastic pain is a label to patients having a predominant central sensitization pain. Our previous Cell Rep study reported that LTP-inducible electrical stimulation (10 V high-frequency or low-frequency) simulate ectopic discharge in spinal dorsal horn (SDH) without significant nerve injury caused nociplastic pain, suggesting that central sensitization can directly cause chronic pain [6]. However, this model still bears a certain degree of skin and muscle damage. Based on these, it is necessary to establish a general and suitable animal model to study the pathogenesis of CPP as well as new drugs to control it.

Ketamine, a general anesthetic agent and N-methyl-D-aspartate receptor (NMDAR) antagonist, represents a promising modality for the management of perioperative pain and refractory chronic pain [7]. However, the well-known psychoactive action, cognitive impairment and neurotoxicity restrict the clinical usefulness of ketamine. Besides, ketamine and its metabolites have rapid-acting antidepressant effects [8–10]. Ketamine metabolite 2R, 6R-HNK stands out to be putative rapid antidepressant drug candidate [11]. Compared with ketamine, 2R, 6R-HNK produces nearly non-existent ketamine-related side-effects [7, 10] due to its low NMDAR-binding affinity [12–14]. Thus, huge interest has grown regarding 2R, 6R-HNK. More surprisingly, several recent lines of evidence have reported its potent analgesic actions when administered i.p. or intranasally in animal models of acute/chronic inflammatory and neuropathic pain [15–18]. However, the mechanism of 2R, 6R-HNK on pain relief has still remained a mystery. Considering the analgesic and central inhibitory effects of 2R, 6R-HNK, its application and development prospects in CPP are worth exploring.

In the present study, we reported that long-lasting mechanical, thermal hypersensitivity and comorbid anxiety, depression and cognitive disorders in a new CPP mouse model induced by LF-PENS. Combining in vivo experiments and in vitro calcium imaging in cultured DRG neurons, we investigated the role of i.t. administration of 2R, 6R-HNK in this model, and explored the possible molecular targeting mechanism. In general, our present study not only established a new noninvasive animal model of CPP, but also provided important insights into the analgesic effects of intrathecal 2R, 6R-HNK and its mechanism.

## Materials and methods

### Animals

C57BL/6 female mice 7–8 weeks old (Guangdong Medical Laboratory Animal Center, China) were pre-habituated to animal facilities (a 12/12 light dark cycle, 23 ± 1 ℃, and ad libitum food and water) for one week prior to experimental and behavioral testing. Previous evidences have shown that ketamine exerts enhanced antidepressant actions in female rodents compared to males [19, 20]. In addition, 2R, 6R-HNK in the brains of female mice is approximately three times higher compared to that of males when the levels of ketamine and norketamine were equivalent [10]. Therefore, only female mice were chosen in this study. Excluding cell experiments, a total of 220 mice were used in the in vivo experiments. The animals used in different experimental processes are shown in Table 1. Some animals that violated the guidelines of tests were excluded from results (e.g., showed no interest in any object during the New-object recognition test). All the behavioral tests were performed by two experimenters, one blinded to drugs and/or surgical treatments. The experimental protocols and animal handling procedures were approved by the Institutional Animal and Use Committee (IASUC), Sun Yat-sen University (Nos. SYXK (yue) 2017–0081 and 2022–0081).

**Table 1:**
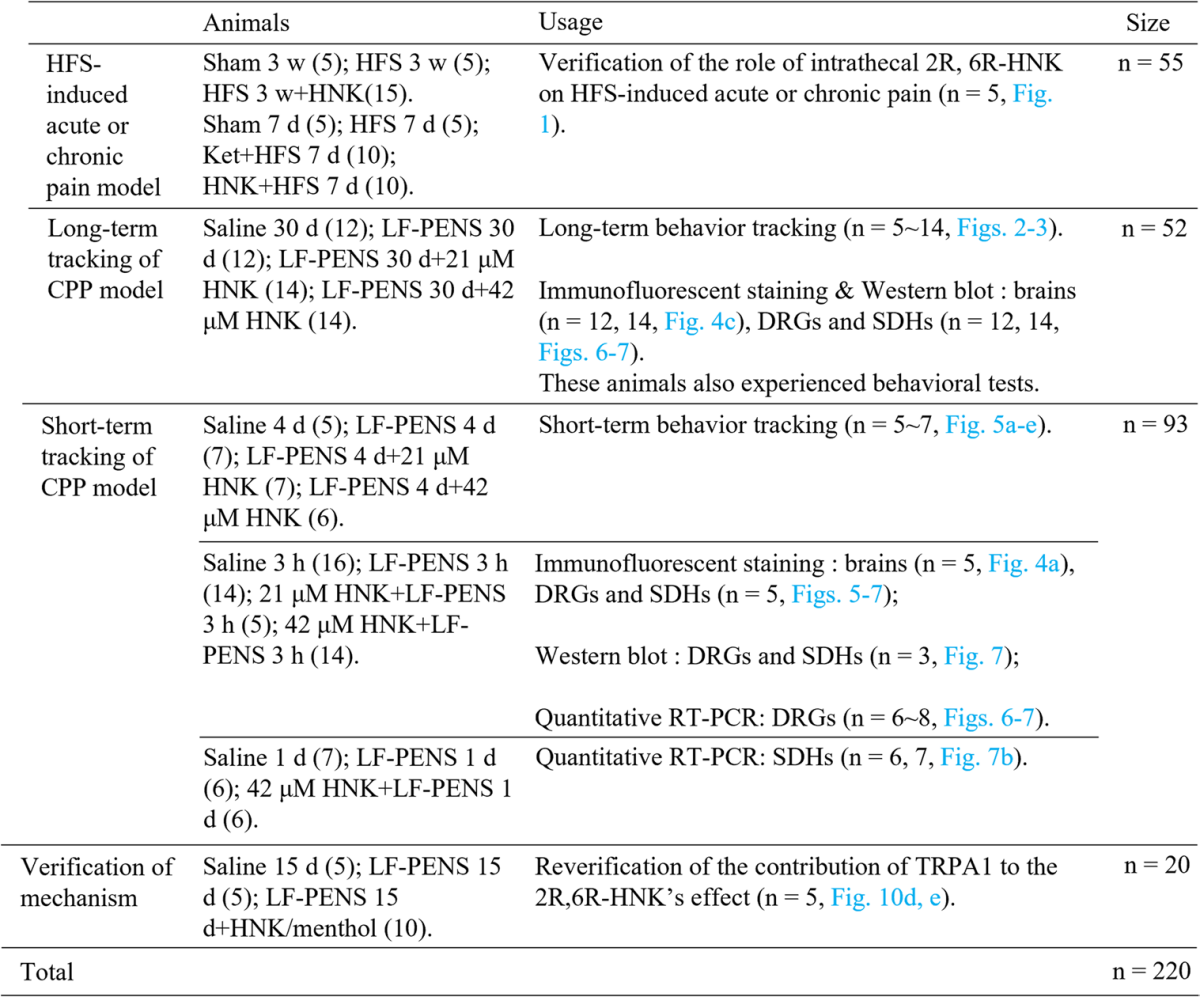
The animals used in different experimental processes

### Drug administration

The drugs included 2R, 6R-HNK (SML1873, Sigma, USA), S-Ketamine (Esketamine, Jiangsu Hengrui Medicine Co., Ltd, China), formaldehyde (252,549, Sigma-Aldrich, USA), menthol (M2772, Sigma), Allyl-isothiocyanate (HE-3277, Beijing Henghui Bio Co., China), capsaicin (21,750, Sigma), capsazepine (GC17918, Glpbio, USA), KCL (P816354, Macklin, China) or vehicle (saline in vivo or HHBS in vitro).

It has been reported that i.p. injection (10 mg·kg^−1^) of 2R, 6R-HNK also turn out to be more effectively analgesic than ketamine in nerve-injury neuropathic pain, postoperative pain and CRPS pain models in preclinic study via a μ-opioid receptor-independent pathway [18], making it a putative anodyne candidate either. Besides, studies have shown that 2R, 6R-HNK concentrations in plasma and brain tissue rapidly peak within 30 min after intravenous (i.v.)/per os (p.o.) administration and then decline fast, which is similar to the liver metabolic pharmacokinetic properties of Ketamine and its high lipid solubility [14, 21]. Based on the previous study, 7 ~ 21 μM i.t. injection of 2R, 6R-HNK is equivalent to i.p. injection of 10 mg·kg^−1^ [22], which is also the concentration for antidepressants [10, 23]. Therefore, to figure out the inhibitory effect of 2R, 6R-HNK on spinal nociception on CPP mouse model, we not only examined the antinociceptive effects of i.t. (7, 21 and 42 μM) and traditional i.p. (10 mg·kg^−1^) administrations, but also compared their actions with S-Ketamine. As for the calcium imaging and cell cultures, our medicine timing and dosage referred to previous literature [24, 25] and were described in detail below.

Adult female mice (20 ± 2 g) were anesthetized with 1.5% isoflurane and injected intrathecally with different drugs in a volume of 10 μl with a Luer-Tip Hamilton syringe at the level of the pelvic girdle. Given that cerebrospinal fluid volume was about 200 μl in each mouse, intrathecal drugs were formulated to 20 times the working concentration in saline, divided and stored at -20 °C before injection. Menthol (420 μM i.t.; 300 μM in vitro) was conserved in equal proportion as effective 2R, 6R-HNK concentration (42 μM i.t.; 30 μM in vitro) from vitro to vivo.

### HFS-induced acute and chronic pain model

Protocols of surgical preparation for HFS-induced acute (within 7 days) and chronic (more than 2 weeks) pain model in mice have been described in our previous study [6]. Briefly, mice were anesthetized with 2% isoflurane anesthesia and the left sciatic nerves were dissected for electrical stimulation with Pt electrodes. HFS at 10 V (HFS: 100 Hz, 0.5 ms, 100 pulses given in 4 trains of 1 s duration at 10 s intervals) was delivered to the left sciatic nerve. In the sham group, only the sciatic nerve was exposed. At the end, the muscle and skin were sutured in two layers.

### LF-PENS-induced acute and chronic pain model

To improve HFS-induced acute and chronic pain model without skin and muscle injuries, we chosen low-frequency percutaneous electric nerve stimulation (2 Hz, 0.5 ms, 10 V, a total of 120 pulses within 1 min) to induce acute and chronic pain model for CPP. Under 2% isoflurane anesthesia, the back skins of mouse left thigh were shaved and LF-PENS was percutaneously delivered to the popliteal fossae with Pt electrodes. Saline controls were only shaved after anesthesia.

### Mechanical sensitivity (von Frey) test

Mice had been continuously acclimated to the test environment and the experimenter for 0.5 h since 3 d prior to formal test. To test mechanical allodynia, mice were placed in the 10 × 10 × 10 cm^3^ isolation rooms on elevated wire grid for 0.5 h to be peaceful but not sleepy. A set of von Frey filaments (0.04–1.4 g; North Coast medical, USA) was used to mechanically stimulate the plantar surface. Each filament was applied vertically to the lateral and medial plantar surface of the paw, and withdrawal response evoked in up to 5 s/at least one of five repetitive stimuli was positive. Mechanical paw withdrawal threshold (PWT) was determined using the up-down method. Mice with pre-surgery/treatment basal threshold (Bas) less than 0.6 g were excluded [26]. We used analgesic efficiency to evaluate the inhibition efficiency of the drug. Rate of change in mouse PWT = (ln2ln(PWT))/(ln2 - ln0.02) × 100, analgesic efficiency = (PWT change rate in model group—PWT change rate in treated group) / PWT change rate in model group × 100.

### Open field test (OFT)

The mice had been given pre-adaption for 0.5 h per day in a 40 × 40 × 40 cm^3^ open acrylic box in the last 3 d before test. During the formal experiment, mice were put in the center of the open field or facing the wall. Their tracks, average speeds and times both in the central (30 × 30 cm^2^) and peripheral area within 10 min were recorded and analyzed with the tracking software.

### Hargreaves test

Hargreaves test was measured by the Plantar Test Apparatus (390G, IITC Life Science, USA). Mice had been given pre-adaption in single home cages about 15 × 15 × 15 cm^3^ for 1 h per day since 3 d before test. Radiation intensity was adjusted to 30, so that the heating temperature reaches about 52 ~ 55 °C within 20-s-trial [27]. In the formal experiment, the mice were placed in the isolation rooms on glass for 0.5 h. With the aiming spot of the transmitter aiming at the middle of foot, the irradiation was turned on, and the latency of shrinking, lifting, licking feet, or jumping was recorded (no response during trials was recorded as 20 s). The test would be repeated 3 times on each rodent foot after no-less-than-5-min interval.

### Tail-flick test

To further evaluate CPP thermal pain, tail-flick test, a commonly used measure of nociception in animals [28], was also performed in this study. The positive tail withdrawal is not only a spinal reflex, but also involved higher brain centers [29]. According to a previous study [26], the cylinder limiter (50 ml) was used to limit mice in size with their tails exposed. Mice were acclimated to the limiter prior to formal test until not be obviously resisting to enter and struggling inside. In the formal experiment, the limiter was held in hand and closed by thumb, leaving the tail hang down naturally. 1/3 to the distal of the tail was drown into the thermostatic water bath (50 °C), and the latency to evoked tail flick reaction within 15 s was recorded (not-reaction was counted as 15 s). The experiments were repeated for 3 times at each temperature with an interval of more-than-1-min, and the tails were wiped until dry at the end of each test.

### Acetone test

After 3 d of continuous acclimatization, mice were placed in the 10 × 10 × 10 cm^3^ isolation rooms on elevated wire grid for 0.5 h to be peaceful but not sleepy. Then, acetone in syringe was sprayed into the plantar surface of each mouse through blunt-plastic-needle. The latency of reaction and corresponding behavioral score were recorded within 3 repeats between no-less-than-1-min interval (foot lifting/shaking/shaving counted 1 while foot licking counted 2, and 1 additionally counted for behavioral continuity) [26, 30].

### Spontaneous pain behavior test

After acclimatization, mice were placed in single transparent home cages about 15 × 15 × 15 cm^3^ to record the spontaneous behaviors during 20-min-exploration/40-min-resting period. According to a previous study [31], spontaneous pain related behaviors of mice were recorded and analyzed (foot retraction/ lifting counted 1 and licking counted 2).

### Sucrose preference test (SPT)

Before SPT, mice were habituated to water deprivation but food *ad libitium* for 24 h (from 8:00 PM on the 7^th^/20^th^ d after LF-PENS to 8:00 PM on 8^th^/21^th^ d). The purpose of water deprivation is to increase the times and total amount of liquid intake within 24 h of the test, improve the difference among groups, and try to avoid the error caused by insufficient intake. For the next 24 h, the mice were placed in individual compartments (15 × 15 × 15 cm^3^) separated by 0.5 × 0.5 cm^2^ wire in plastic cages with adequate food and bedding materials, so that the mice could sense and communicate with their companions. Distilled water and 2% sucrose solution were loaded in pairs of water feeder separately. The bottles of sucrose solution/water were weighed before and after the later 24-h test to calculate liquid consumption. According to the previous study [27], preference was defined as a percentage of sucrose intake to total volume consumed during trials.

### Tail suspension test (TST)

Mice were hanging over with tail stuck by medical tape at 1 cm to the distal and body naturally hanged down, and the distance from the fall protection is not less than 50 cm. Within 5 min their struggling and stable time were recorded on a white background. Mice were also recorded as stationary due to inertial swing. If a mouse successfully escaped by grasping its tail, the animal will be excluded [32, 33].

### Forced swimming test (FST)

The FST was performed in a transparent glass beaker with inner diameter of 30 cm, containing water about 25 °C. Water was changed between rodents and water level was no less than 20 cm for bottom contactless [27]. Animals would have 10 min swim-adaption 3 d before test, and be forced swimming for 5 min in formal trials. All mice would dry by electric fire and their behaviors in test were recorded directly above the containers. The videos were then analyzed by tracking software.

### New-object recognition test (NORT)

We performed NORT based on a previous study [34]. Since 3 d before test, the mice had been given pre-adaption for 0.5 h per day in a 40 × 40 × 40 cm^3^ open acrylic box, and objects A and B with limited edges and corners (balls and cylinders) were selected as new objects for identification. In the formal experiment, two identical objects A were fixed in the central area of the box about 10 cm apart, and mice were put into the box in turn facing the wall. Combined with the trajectory tracking software, the movement of mice within 5 min and their interacting time with two objects were recorded (included sniffing, pushing, climbing and other behaviors, and single interaction time less than 1 s was counted as 1 s). After taking out the mice, the ones who spent more time on were reserved, while the other were replaced with new objects B. The mice were put into the box once again facing the box wall, and so did the recorder work. The box would be wiped with 70% ethanol solution after each trial. Interacting time to objects A and B within the latter 5 min were analyzed to calculate the new object preference, which was defined as a percentage of B-interacting time to total interaction during trials.

### Elevated plus maze test (EPMT)

In this experiment, pre-adaption was abandoned to get rid of bias caused by memory. Mice were put in the center of a plus maze (each arm 30 × 5 cm^2^), elevated one meter above the floor with two face-to-face open arms and two closed arms (with 20 cm-tall walls on both sides) at less than 100 lx. Each mouse was heading to open arms at first and recorded with videotapes in 5 min. The plus maze would be wiped with 70% ethanol solution after each trial. The times of rodents’ entrance of different arena [35] were evaluated and statistically analyzed.

### Immunofluorescent staining (IF)

Mice were perfused intracardially with 20 ml PBS (4 °C, pH = 7.4) followed by 20 ml 4% paraformaldehyde (PFA, 4 °C; Sigma) in PBS. Brains, L4 DRGs and L4 ~ 5 spinal cord were harvested and post-fixed for 4 ~ 6 h. After dehydration with 30% sucrose in PBS at 4 °C, all the tissues were sliced into 25 μm (brain),16 μm (DRG),18 μm (spinal cord) sections using a cryotome (CM3050S, Leica, Germany), transferred on to Superfrost Plus Microscope slides (FD Neuro Technologies, Inc, USA). After 3 washes in PBS, the cultured DRG neurons on coverslips were fixed with 4% PFA for 30 min and then washed in PBS for another 3 times without dehydration. Next, the slices/neurons were blocked with 5% donkey serum in 0.3% Triton X-100 (Sigma) for 60/30 min at room temperature (RT), and then incubated for 18 h on cradle at 4 °C with a mixture of primary antibodies: rabbit anti-c-Fos antibody (1:500, 2250S, Cell Signaling Technology (CST), USA), rabbit anti-p-ERK antibody (1:500, 4370, CST), mouse anti-GFAP antibody (1:500, 3670, CST), goat anti-Iba1 antibody (1:1000, ab5076, Abcam, USA), goat anti-CGRP antibody (1:1000, NBP3-00520, Novus, USA), mouse anti-CGRP antibody (1:2000, ab81887, Abcam), rabbit anti-TRPA1 antibody (1:500, SAB2105082, Sigma), rabbit anti-TRPV1 antibody (1:1000, GTX54762, Genetex, USA), mouse anti-VGLUT2 antibody (1:100, MA5-27,613, Invitrogen, USA). Following 3 washes, the sections were then incubated with secondary antibodies (Alexa Fluor 488, 555, 647; Life Technologies, USA) at RT for 60 min and rinsed for another 60 min. The slices were protected by Antifade Mounting Medium with DAPI (P0131, Beyotime, China) under coverslips and fluorescent images were obtained with a fluorescence microscope (EVOS FL, Thermo Fisher Scientific, USA).

The ranges of nuclei of interest were determined by mouse brain atlas (for example, the ACC was 0.62 ~ 0.14 mm before bregma and other brain nuclei were 1.46 ~ 1.94 after bregma). 5 mice were used for analysis in each group, and 4 slices with strong positive signal within the limited range of the nuclei (Fig. 4a, red boxes) were blindly selected from the 6–10 stained ones in each group for statistics. The total number of c-Fos, *p*-CREB, *p*-ERK and other positive immunoreactive cells were calculated in each section by ImageJ software (National Institutes of Health, Bethesda, MD). According to the previous study [6], the intensity of the positive immunoreactivity was quantified using relative optical density (RelOD) by the ImageJ image processing. The intensity in sample from sham, saline or control tissues was set as 1 or 100% baseline. The IF quantitative statistical methods in L4 DRGs and L4 ~ 5 SDHs were the same as above.

### RT-qPCR

L4 DRGs, L4 ~ 5 SDHs or cultured DRG neurons were extracted and homogenized in RNAzol® RT (RN190-200, MRC, China). RNA was isolated using RNAzol/double-free water extraction and cDNA was prepared from total RNA by reverse transcription reaction with Evo M-MLV RT Premix (AG11706, Accurate Biology, China). qPCR was performed with CFX 96 touch (C1000™, Bio-rad, USA) using 2 × Master qPCR Mix SYBR Green I (TSE201, TSINGKE, China) in following conditions: 95 °C for 30 s; 40 cycles of 95 °C for 5 s, 60 °C for 30 s and melting analysis at last. Primers sequences were as follow (Table 2), and relative mRNA expressions were normalized to *Actb* in each group and the ratios from saline/sham/control group were set as baseline 1.

**Table 2:**
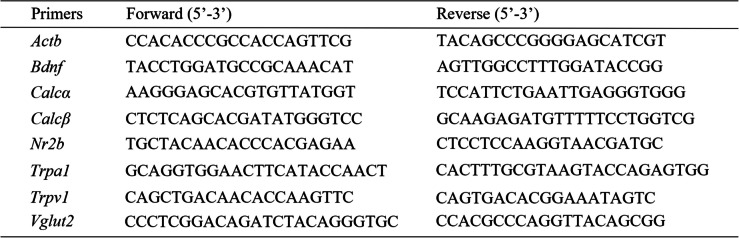
Primers for RT-qPCR in tissues and cells

### Western blot (WB)

Mice were subjected to deep anesthesia by urethane, followed by transcardial perfusion with cold PBS. Ipsilateral L4 ~ L5 DRGs and SDHs were carefully dissected, homogenized, and sonicated in a lysis buffer containing a protease inhibitor cocktail (Cat# P1045, Beyotime Biotechnology). For protein analysis, 15 μg total proteins were loaded into each well, separated using SDS-PAGE, and subsequently transferred to PVDF membranes. The membranes were blocked with a solution of 5% bovine serum albumin in TBST (Tris-buffered saline, 0.1% Tween 20) at RT for 1 h. Next, they were incubated overnight at 4 °C with primary antibodies including Rb anti-*p*-ERK (#4370, Cell Signaling Technology, 1:1000), Rb anti-TRPA1 (SAB2105082, Sigma, 1:1000), Rb anti-TRPV1 (GTX54762, Genetex, 1:200, and Ms anti-β-actin (ab170325, Abcam, 1:1000). Subsequent steps involved incubating the blots with horseradish peroxidase-conjugated IgG at RT for 1 h. Protein bands were detected by Omni-ECL™Femto Light Chemiluminescence Kit (SQ201, Epizyme) and then captured by Tanon-5200 Chemiluminescent Imaging System (Tanon Science and Technology, China). The integrated optical density of each immunoreactive band was measured Software ImageJ 1.51j8, and then normalized to β-actin. The protein X/β-actin ratio in the saline group was established as the baseline for comparison.

### DRG neuronal culture

3-week C57BL/6 female mice (Guangdong Medical Laboratory Animal Center, China) were made unconscious under isoflurane anesthesia (5%) and then decapitated immediately. All the DRGs were dissected out, cut into pieces and transferred to DMEM/F12 (C11330500BT, GIBCO, USA). The samples were digested with 5 mL DMEM/F12 containing collagenase (3 mg, C9891, Sigma) and trypsin (2 mg, T9201, Sigma) for 20 ~ 25 min and then the same volume of complete medium (DMED/F12 + 10% FBS + 1% penicillin and streptomycin (100 × , GIBCO)) was added to stop the digestion. Next, the samples were filtrated (100 μM, BS-100-CS, Biosharp, China) and centrifuged for twice (1000 g × 5 min, 20 °C), and then resuspended into single cells.

For calcium imaging, these cells were cultured in 0.01% PLL-coated (Poly-L-Lysine, P4832, Sigma) 96-well plates in complete medium in humid conditions and 5% CO_2_ at 37 °C for 6 h. For TRPA1 agitation test in vitro, DRG neurons were incubated in coated 24-well plates for 72 h in complete medium containing 2% B-27 Supplement 50 × (17,504–044, GIBCO), which were renewed every day. 5 μg /mL Ara-C (C2035, TCI, China) was added at 24 h to remove irrelevant cells [36]. The time course and concentration of 2R, 6R-HNK (30 μM, at 48 h), menthol (300 μM, at 48 h), capsazepine (10 μM, at 48 h + 30 min) and formaldehyde (10 μM, at 48 h + 30 min) referred to our results or previous research [36].

### Calcium imaging

DRG cells were incubated with Fluo-8 Calcium Flux Assay Kit (ab112129, Abcam) in 37 °C CO_2_ incubator and RT for 30 min without light exposure, and then fluorescence dye was rinsed and replaced with 50 μl HHBS (CB1048, G-CLONE, China) [24, 25]. Then, calcium imaging was carried on Automatic High-pass Living Cell Imaging Analysis System (lionheart FX, BioTek, USA) in the following conditions: 37 °C, 5% CO_2_ in dark, with cylinders of Auto Sampler filled with 10 × drugs, containing 1 mM Allyl-isothiocyanate (AITC, an agonist of TRPA1 receptor, 100 μM, 120 or 30 s), 10 μM capsaicin (Cap, an agonist of TRPV1 receptor, 1 μM, 30 s) and 1.44 M KCL (a broad agonist of neurons, 144 mM, 30 s). Cells turned out to be overlapping or losing of integrity, as well as floating during experiments were excluded. To avoid interference from inactive/dead cells, the DRG neurons with calcium activity in response to KCL were used for statistical analysis. Besides, those turned out to be inactive (ascending average fluorescence intensity/baseline < 10%) to both AITC/Cap and KCL were classified as meaningless.

In details, blank control was added with HHBS, and the experimental group was pre-incubated by 2R, 6R-HNK (3, 10, 30 μM in HHBS) for 3 h before being put on the machine. Ex/Em = 490/525 nm, and the baseline was recorded for 15 s at 3 s/sheet, followed by AITC or caspaicin stimulation, and recorded for 15 s at 30 ms/sheet. Finally, cells were recorded for another 30 s at 30 ms/sheet after 2^nd^ stimulation from KCL or capsaicin. The interval between administrations is 2 min/80 s. Fluorescence intensity was analyzed and statistically analyzed using Graphpad Prism.

### Statistical analysis

All data were presented as mean ± SEM. Statistical analysis was calculated using GraphPad Prism Version 8.3.0 (GraphPad Software, LLC, CA, USA). Level of significance was set at *p* < 0.05. Kruskal–Wallis non-parametric test (followed by Dunn's multiple comparisons test to establish significance) was applied to the data that do not pass the homogeneity of variance test or not follow the normal distribution. When the data in groups were normally distributed with equal variances, t-test (2 tailed) was performed between 2 groups or ANOVA (followed by Tukey’s or Bonferroni's) for more groups. Behaviors results containing time and group two factors were tested by two-way repeated ANOVA (followed by Tukey’s). Detailed statistical analysis is summarized in Supplementary Table 1 (Table S1).

## Results

### Single intrathecal 2R, 6R-HNK produces delayed antinociception in HFS-induced acute and chronic pain

**Fig. 1:**
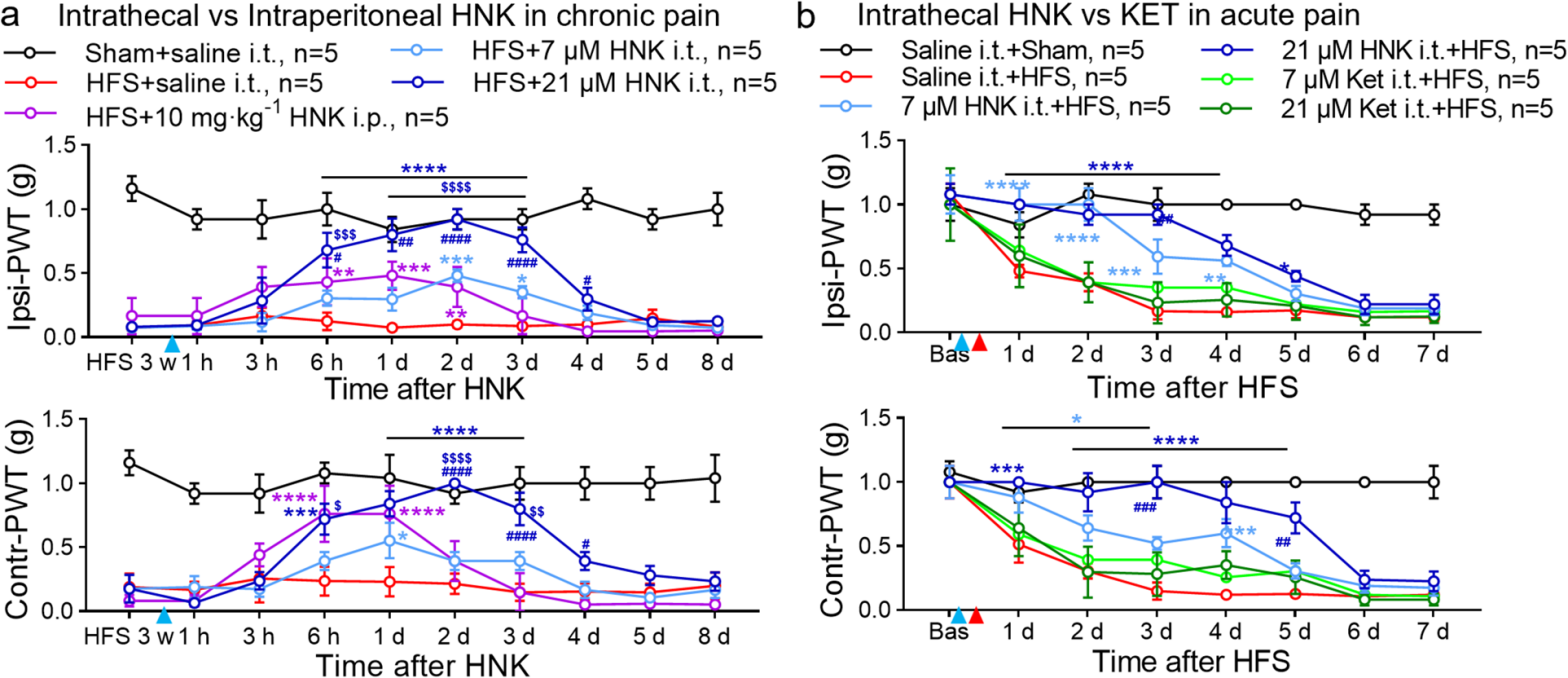
Intrathecal 2R, 6R-HNK exerts delayed antinociceptive effects on HFS-induced acute and chronic pain. **a** Intrathecal (i.t. 7, 21 μM, 10 μl) or intraperitoneal (i.p. 10 mg·kg^−1^) 2R,6R-HNK (HNK) reversed the reduction of the ipsilateral (Ipsi-) or contralateral (Contr-) paw withdrawal threshold (PWT) at 3 w after 10 V high-frequency-stimulation (HFS) of left sciativ nerve (*n* = 5 mice/group). Blue triangles indicated the time point of saline or drugs treatment ( the same in the follows). **b** Intrathecal preadministration of 2R, 6R-HNK rather than S-Ketamine (Ket) at the roughly same dosages (7, 21 μM) delayed HFS-induced acute mechanical pain. 2R, 6R-HNK, S-Ketamine or saline was intrathecally delivered 30 min before HFS. Red triangles showed the time point of sham operation or HFS delivered to sciatic nerve. ^*^p < 0.05, ^**^p < 0.01, ^***^p < 0.001, ^****^p < 0.0001 versus the HFS + saline i.t. or Saline i.t. + HFS group; ^#^*p* < 0.05, ^##^*p* < 0.01, ^###^*p* < 0.001, ^####^*p* < 0.0001 versus the HFS + 10 mg·kg^−1^ HNK i.p. or 7 μM Ket i.t. + HFS group; ^$^*p* < 0.05, ^$$^*p* < 0.01, ^$$$^*p* < 0.001, ^$$$$^*p* < 0.0001 versus the HFS + 7 μM HNK i.t. or 7 μM HNK i.t. + HFS group

To investigate the actions of 2R, 6R-HNK in CPP, we firstly evaluated its analgesic efficiency in 10 V HFS-induced acute and chronic pain model [6] by two administration routes: common i.p. (10 mg·kg^−1^) and innovative i.t. (7, 21 μM) injections. Consistent with our previous study [6], the mechanical PWT significantly decreased in HFS pain model (Fig. 1a, b). A single i.p. 2R, 6R-HNK (10 mg·kg^−1^) slightly reversed the reduction of ipsilateral- and contralateral-PWT (Ipsi-PWT & Contr-PWT) by HFS from 6 h to 2 d (Fig. 1a). We also observed a mild analgesic effect of 7 μM intrathecal 2R, 6R-HNK at 2 ~ 3 d. Strikingly, intrathecal 2R, 6R-HNK at high dose of 21 μM was more effective than that of 7 μM low dose or even equal concentration of i.p. 10 mg·kg^−1^ intraperitoneal injection in the treatment of mechanical pain (from 6 h to 4 d). The strongest antinociceptive efficiency of i.p. 2R, 6R-HNK was at 1 d after administration, while that of i.t. 2R, 6R-HNK was at 2 d. A similar analgesic action also has been observed on the contralateral side. All the data suggested that i.t. 2R, 6R-HNK produced a dose-dependent and delayed antinociception on HFS-induced nociplastic pain, which is more effective and persistent than the i.p. dose (10 mg·kg^−1^ equal with i.t. 21 μM). Due to the use of S-Ketamine for preclinical and clinical analgesia [37, 38], we also compared isodose i.t. 2R, 6R-HNK with S-Ketamine in preventing HFS-induced nociplastic pain (Fig. 1b). S-Ketamine (7, 21 μM) had no effect on the development of mechanical allodynia by HFS. Surprisingly, both doses of intrathecal 2R, 6R-HNK markedly inhibited the early induction of bilateral mechanical hypersensitivity in dose-dependent manner. Together, intrathecal 2R, 6R-HNK owned a delayed antiallodynic effect in acute and chronic nociplastic pain.

### Multiple intrathecal 2R, 6R-HNK alleviates chronic pain induced by LF-PENS

**Fig. 2:**
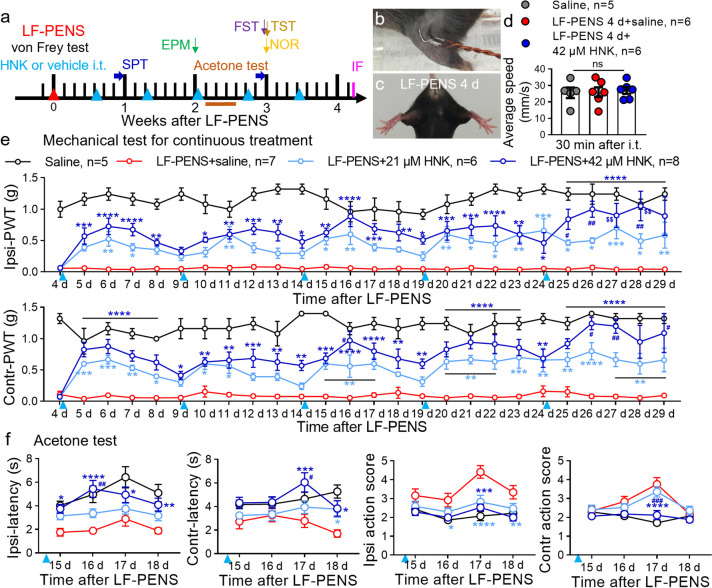
Multiple intrathecal 2R, 6R-HNK provides a sustained analgesic effect on chronic pain induced by LF-PENS. **a** Schematic overview of experiments for intrathecal injections of 2R, 6R-HNK (21, 42 μM, 10 μl, since 4 d after LF-PENS, at 5-days intervals) or saline and a variety of behavioral tests in LF-PENS-induced chronic pain model. OFT, open field test; EPM, elevated plus maze test; SPT, sucrose preference test; FST, forced swim test; TST, tail suspension test; NOR, new object recognition test; IF, immunofluorescence. Blue or red triangles indicated the time point of drugs/saline or LF-PENS treatments. As the same in the follows (*n* = 5 ~ 8 mice/group). **b** Representative images of LF-PENS (2 Hz, 0.5 ms, 10 V, a total of 120 pulses for 1 min) of the left popliteal fossa. **c** Tail suspension test showed normal hind-limb splay at 4 d after LF-PENS. **d** No difference of the walking speed was noticed within 10 min among the groups. **e** The time course of Ipsi- or Contr-PWT to respond to mechanical stimulus following LF-PENS with multiple intrathecal treatments of saline or 2R, 6R-HNK (21, 42 μM). **f** 2R, 6R-HNK dose-dependently reversed LF-PENS-induced cold hyperalgesia. Acetone tests were performed at 15–18 d after LF-PENS. ^*^p < 0.05, ^**^p < 0.01, ^***^p < 0.001, ^****^p < 0.0001 versus the LF-PENS + saline group; ^#^*p* < 0.05, ^##^*p* < 0.01, ^###^*p* < 0.001 versus the LF-PENS + 21 μM HNK group; ^$$^*p* < 0.01 versus Day 8^th^ or 9^th^ after LF-PENS

Since HFS-induced chronic pain model still has a certain degree of skin and muscle damages, we established LF-PENS-induced chronic pain model by low-energy stimuli (2 Hz, 0.5 ms, 10 V, a total of 120 pulses for 1 min) in the left popliteal fossa (Fig. 2b). A variety of behavioral techniques were used to evaluate the changes of pain, mood and cognition of this model, and the therapeutic effects of 2R, 6R-HNK (7, 21, 42 μM) on the model were also examined (Fig. 2a). TST and OFT showed that the model mice exhibited normal hind-limb splay and motor function at 4 (Fig. 2c, d), 7 or 21 d (data not shown) after LF-PENS as compared to saline group, suggesting no significant tissue injury. Pain behavioral tests demonstrated that LF-PENS-induced model mice displayed bilateral mechanical hypersensitivity (Fig. 2e) and cold allodynia (Fig. 2f) lasting for 3 ~ 4 weeks. Overall, LF-PENS induced a noninvasive chronic pain model.

Considering our above results showing that a single intrathecal 2R, 6R-HNK had an antinociceptive effect lasting 4 days (Fig. 1), we utilized multiple intrathecal administration with a 5-day interval to assess the therapeutic effect of 2R, 6R-HNK on LF-PENS model (Fig. 2a). To be mentioned, 2R, 6R-HNK did not affect the velocity of movement in OFT at 0.5 h after intrathecal administration, indicating no sedative effect at the tested dosage (Fig. 2d). Unlike 7 μM dosage (data not shown), sequential intrathecal 2R, 6R-HNK (21, 42 μM) from 4 to 29 d after LF-PENS elevated the decreased PWT (Fig. 2e). Interestingly, 2R, 6R-HNK in the LF-PENS model reached the strongest analgesic response at the 2^nd^ d after each dose, similar with that in the HFS model. Moreover, multiple intrathecal administration did not reduce the analgesic benefit of 2R, 6R-HNK. Instead, the 5^th^ application of 42 μM 2R, 6R-HNK produced a stronger analgesic effect on day 4 or 5 than the 1^st^ treatment (day 28 vs. 8, day 29 vs. 9 after LF-PENS). In addition, 2R, 6R-HNK also alleviated bilateral cold pain sensitivity in a dose-dependent manner (Fig. 2f). Collectively, intrathecal 2R, 6R-HNK at therapeutic doses did not display analgesic tolerance, but rather a superimposed effect.

### Repeated intrathecal 2R, 6R-HNK relieves the LF-PENS-induced depression and cognitive dysfunction, but not anxiety

**Fig. 3:**
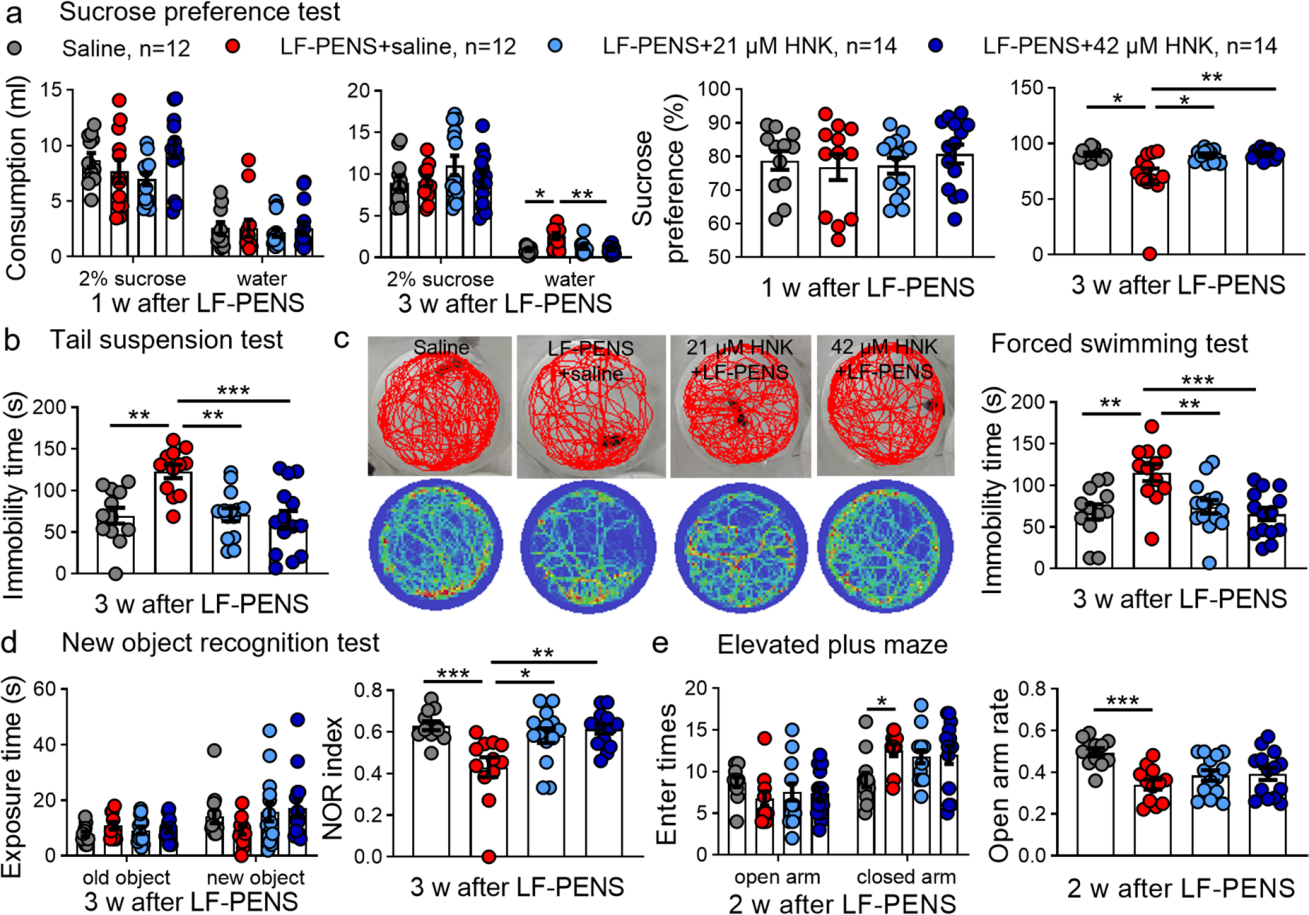
Multiple intrathecal HNK inhibits LF-PENS-induced depression and cognitive deficits, but not anxiety. **a-d** Multiple intrathecal 2R, 6R-HNK administration eliminated LF-PENS-induced changes in sucrose preference test (**a**), tail suspension test (**b**), forced swimming test (**c**), new object recognition test (**d**) at 3-w after stimuli. **e** Both dosages of 2R, 6R-HNK had no effect on anxiety-like behavior at 2-w after LF-PENS (*n* = 12 or 14 mice/group), ^*^p < 0.05, ^**^p < 0.01, ^***^p < 0.001 between groups

To explore whether LF-PENS-induced chronic pain model is applicable to CPP studies, we also synchronically examined behavioral changes in mood and cognition (Fig. 2a). SPT provided that 3-w LF-PENS mice but not 1-w ones displayed higher water intake but lower sucrose preference in 24-h protocols with only water deprivation (Fig. 3a), indicating LF-PENS model displayed progressive anhedonia, a core symptom of depression. Additionally, in TST (Fig. 3b) or FST (Fig. 3c), 3-w LF-PENS mice demonstrated increased immobility time as compared to saline mice. Besides, in NORT, 3-w LF-PENS mice exhibited no significant difference in exposure time to old and new objects, but a decrease in preferential exploration of the new objects compared to saline mice (Fig. 3d). We had also assessed anxiety behavioral by EPMT at 2-w after LF-PENS, and found that the enter rate to the open arm was significantly decreased in model mice (Fig. 3e). In general, our findings demonstrated that the LF-PENS model mice not only develop noninvasive chronic pain, but also present the comorbidities: depression and cognitive deficits at the 3^rd^ week, and anxiety at the 2^nd^ week.

**Fig. 4:**
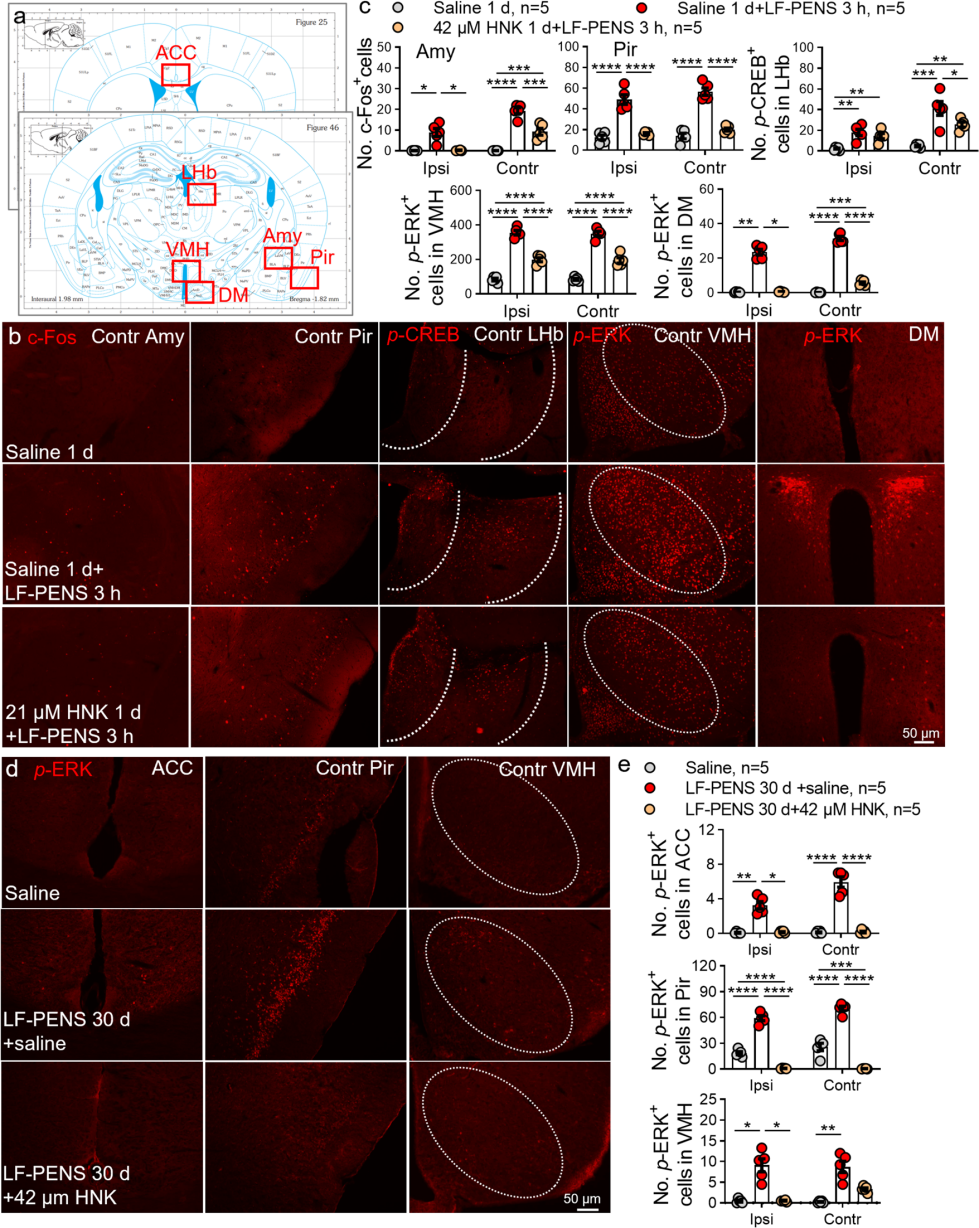
2R, 6R-HNK reverses the activation of several brain nuclei induced by LF-PENS. **a** Schematic diagram of nuclei of interest in brain slices. ACC: Amy: amygdala; Pir: piriform cortex; LHb, lateral habenular nucleus; VMH: ventromedial nucleus of hypothalamus; DM: dorsomedial hypothalamic nucleus. **b-c** Representative images and quantification of the number of c-Fos^+^, *p*-CREB^+^ or *p*-ERK^+^ cells showing that 2R, 6R-HNK pretreatment suppressed the increases in their expression in the bilateral Amy, Pir, LHb, VMH or DM at 3 h after LF-PENS. **d-e** Representative images and quantification of the number of *p*-ERK^+^ cells indicating that multiple 2R, 6R-HNK appliance suppressed the increases of neuron excitability markers’ expression in bilateral ACC, Pir and VMH at 30 d (*n* = 5 mice/group, 4 sections/mouse), ^*^p < 0.05, ^**^p < 0.01, ^***^p < 0.001, ^****^p < 0.0001 between groups

The network of neural circuits associated with mood and stress, which consists of amygdala (Amy) [39], piriform cortex (Pir) [40], lateral habenular nucleus (LHb) [41], anterior cingulate cortex (ACC) [42] and hypothalamus [43], is more susceptible to chronic pain. The various nuclei of the hypothalamus [such as ventromedial nucleus of hypothalamus (VMH) and dorsomedial hypothalamic nucleus (DM)] form a vital hub in this network and play a key role in depressive symptoms [43]. Next, we used three markers of neuronal excitability [c-Fos [44], phosphorylation of cAMP-response-element-binding protein (*p*-CREB) [45] and extracellular signal-regulated protein kinase (*p*-ERK) [46, 47]] to examine the changes in these brain regions (Fig. 4a), which are associated with the above behaviors and clinical CPP [48–55]. Amazingly, we noticed these excitatory signals were enhanced in bilateral Amy, Pir, LHb, VMH and DM at 3 h after LF-PENS (Fig. 4b, c), and even in ACC, Pir and VMH at 30 d (Fig. 4d, e). Accordingly, we preliminarily considered that LF-PENS-induced chronic pain as a newly successful CPP model.

Synchronously, we also investigated the effects of repeated intrathecal 2R, 6R-HNK (21, 42 μM) on mood and cognitive behaviors in this model. Both doses of 2R, 6R-HNK markedly suppressed the increase of water consumption and the decline of sucrose preference at 3-w following LF-PENS (Fig. 3a). Likewise, 2R, 6R-HNK only in high concentration clearly reduced LF-PENS-induced increases of the immobility time in the tail suspension and forced swimming tests (Fig. 3b, c), and prevented the cognitive index NOR decline in LF-PENS mice (Fig. 3d). However, 2R, 6R-HNK at our used concentration had no inhibitory effect on LF-PENS-induced anxious behavior (Fig. 3e). Besides, 2R, 6R-HNK (21, 42 μM) also offered wholly or partly inversion effect on altered brain in CPP model (Fig. 4). Given the above, our preclinical studies suggested that intrathecal 2R, 6R-HNK had an inhibitory efficacy in LF-PENS-induced comorbid depression and cognitive impairments, but not anxiety, which may be related to the inhibition of c-Fos, *p*-CREB and *p*-ERK expression in the involved brain regions.

### 2R, 6R-HNK pretreatment mitigates acute hypersensitivity induced by LF-PENS

**Fig. 5:**
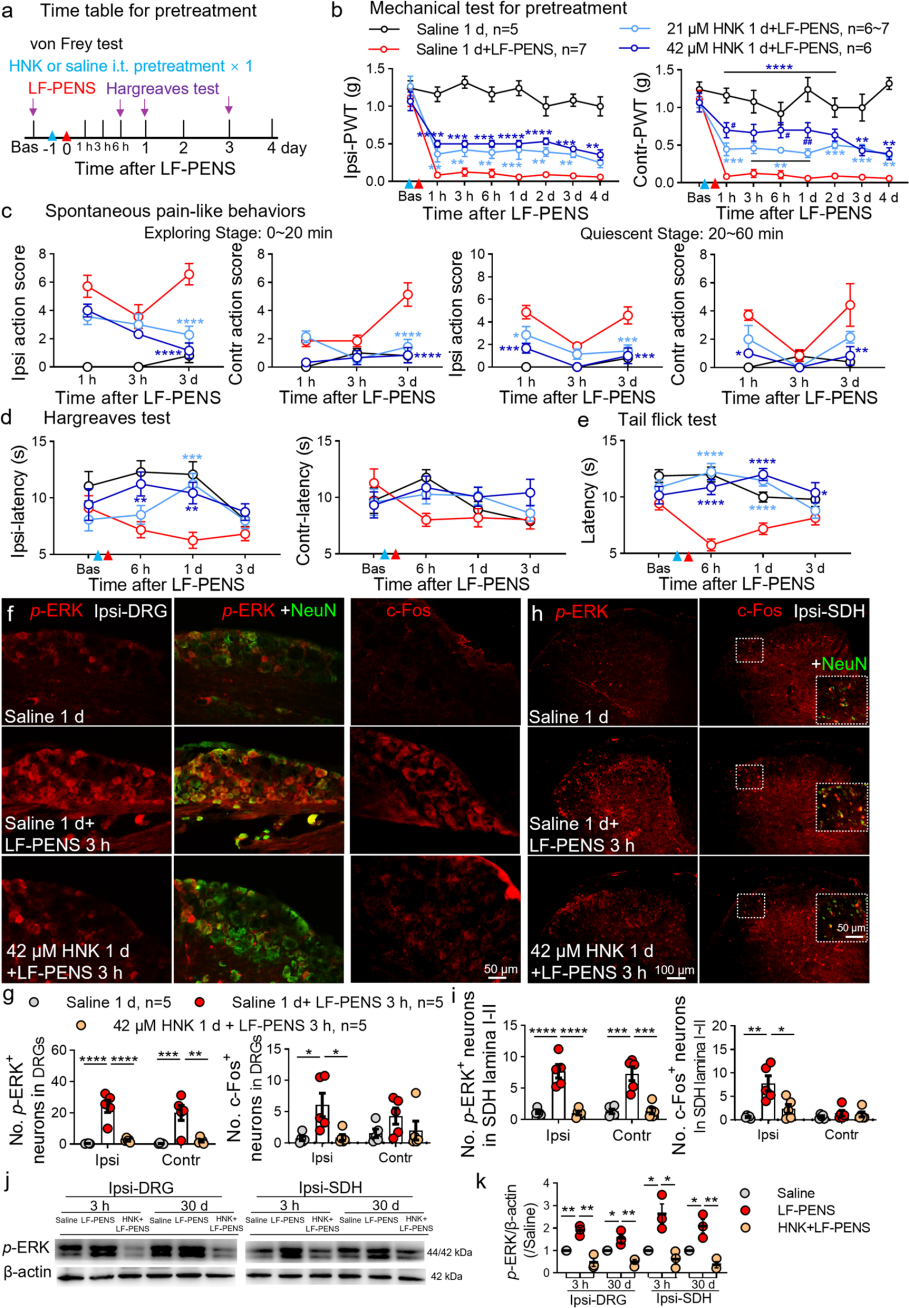
Intrathecal pre-administration of 2R, 6R-HNK alleviates LF-PENS-induced acute pain and neuronal hyperexcitability. **a** Experimental timeline of 2R, 6R-HNK pretreatment on LF-PENS-induced acute pain. 2R, 6R-HNK (21, 42 μM, 10 μl) or saline was i.t. injected 1 d prior to LF-PENS. **b-e** Intrathecal preconditioning of 2R, 6R-HNK reversed LF-PENS-induced behavioral changes in bilateral PWT (**b**), spontaneous pain-related behavioral scores (**c**), and response latencies to Hargereaves test (**d**) and tail-flick test (**e**) (*n* = 5 ~ 7 mice/group), ^*^p < 0.05, ^**^p < 0.01, ^***^p < 0.001, ^****^p < 0.0001 versus the same timepoint of the Saline 1 d + LF-PENS group. **f-i**: Representative images and quantification of the number of *p*-ERK^+^/NeuN^+^ (**f**, **g**) or c-Fos^+^/NeuN^+^ (**h**, **i**) cells showing that 2R, 6R-HNK pretreatment suppressed the increases in *p*-ERK and c-Fos’s expression in neurons of the bilateral DRGs or SDHs 3 h after LF-PENS (*n* = 5 mice/group, 4 sections/mouse). **j**, **k**: Western blot results showing the changes of *p*-ERK expression in the ipsilateral L4, 5 DRGs and SDH at 3 h or 30 d after LF-PENS with or without signal or multiple intrathecal 2R, 6R-HNK (*n*= 3 mice/group), *p* < 0.05, ^**^p < 0.01, ^***^p < 0.001, ^****^p < 0.0001 between groups

To explore the mechanism of 2R, 6R-HNK on CPP, we further evaluated the preventive effects of 2R, 6R-HNK on LF-PENS-induced acute pain (Fig. 5a). Given the delayed effect of 2R, 6R-HNK on pain behaviors from Kroin’s report [18], it was intrathecally performed 1 d before LF-PENS. We observed that a dramatically decrease in bilateral mechanical PWT as early as 1 h after LF-PENS, as well as an increase in spontaneous pain-like behavior during the exploratory and quiet periods (Fig. 5b, c). Similarly, LF-PENS decreased the latencies until response to thermal stimuli in ipsilateral Hargreaves test (Fig. 5d) and Tail-flick test (Fig. 5e). These data demonstrated that LF-PENS induced intense bilateral acute mechanical allodynia, spontaneous pain and thermal hyperalgesia, similar with the effect of HFS we reported previously [6]. As expected, the pretreatment of single intrathecal 2R, 6R-HNK distinctly prevented the decline of bilateral PWT (Fig. 5b) from 1 h to 4 d after LF-PENS. In addition, 2R, 6R-HNK produced clearly antinociceptive efficacy in spontaneous pain-like behavior (Fig. 5c) and ipsilateral thermal hyperalgesia (Fig. 5d, e). In contrast to chronic pain, pretreatment of intrathecal 2R, 6R-HNK had relatively slightly antinociceptive effect on LF-PENS-induced acute pain without significant dose dependency. Subsequently, we also examined the changes of *p*-ERK and c-Fos signals in neurons of DRG and SDH (marked by NeuN). Consistent with the results of pain behaviors,* p*-ERK and c-Fos upregulations in bilateral DRGs and the superficial layer of SDH at 3 h after LF-PENS were also prevented by 2R, 6R-HNK pretreatment (Fig. 5f-i). Besides, Western blot results not only confirmed the changes of *p*-ERK in L4-5 DRGs and SDHs of each group at 3 h after LF-PENS, but also indicated that at 30 d, multiple intrathecal administration of 2R, 6R-HNK also reduced the upregulation of *p*-ERK (Fig. 5j, k). Together, our results supported that a sustained analgesic effect of intrathecal 2R, 6R-HNK probably by inhibiting neuronal hyperexcitability in ascending nociceptive pathway.

### Intrathecal 2R,6R-HNK strictly depresses LF-PENS-induced CGRP upregulation but not microglial activation

**Fig. 6:**
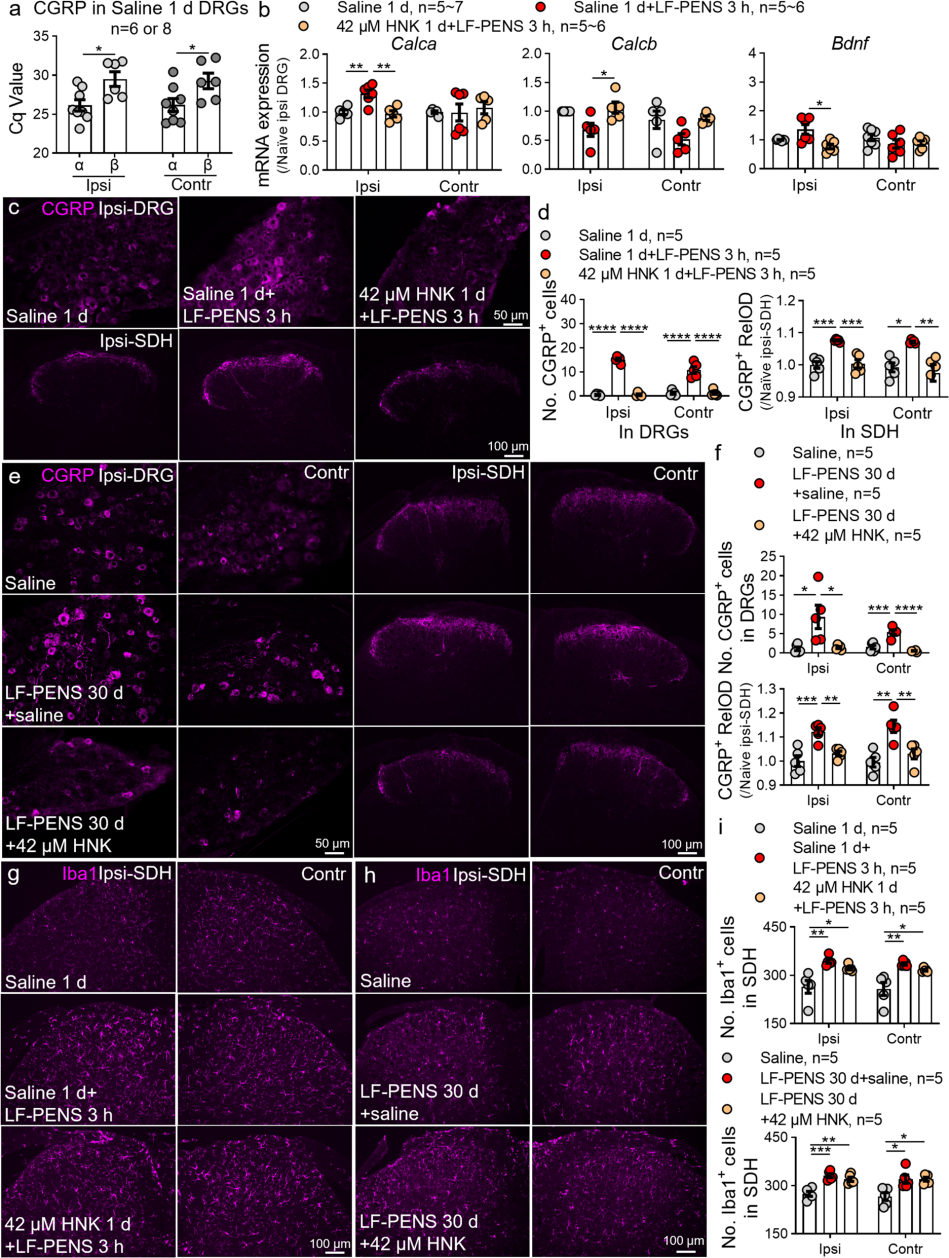
LF-PENS-induced CGRP overexpression rather than microglial activation is suppressed by intrathecal 2R,6R-HNK. **a** PCR cycle quantification (Cq) value of CGRPα mRNA (*Calcα*) was lower than β (*Calcβ*) in Saline 1 d L4 DRGs (*n* = 6 or 8 mice/group). **b** RT-qPCR analysis of *Calcα*, *β*, and *Bdnf* mRNA in bilateral DRGs at 3 h from different groups (*n* = 5 ~ 7 mice/group). **c-i** Representative immunofluorescent images and statistical analysis demonstrated that CGRP upregulation but not microglial activation in bilateral L4 DRGs and L4 ~ 5 SDH at 3 h (**c, g**) or 30 d (**e, h**) after LF-PENS was inhibited by 2R, 6R-HNK (*n* = 5 mice/group, 4 sections/mouse). ^*^p < 0.05, ^**^p < 0.01, ^***^p < 0.001, ^****^p < 0.0001 between groups

Considering the essential role of CGRP in the pathophysiology of migraine (a common CPP) [56] and the correlation of its expression with HFS-induced chronic pain [6], we also quantitatively examined the expression of CGRP in the LF-PENS model and the possible mechanism of 2R, 6R-HNK’s effect. Our RT-qPCR data showed that the cycle quantification (Cq) value of CGRPα (*Calcα*) was lower than β (*Calcβ*) in naïve DRG samples (Fig. 6a), indicating *Calcα* mRNA took predominant role in DRGs. The result was in line with the previous reports [57]. Compared to saline group, LF-PENS indeed caused the upregulation of *Calcα* mRNA in the ipsilateral DRG at 3 h after stimuli (Fig. 6b) rather than *Calcβ* or brain-derived neurotrophic factor (*Bdnf*) mRNA. Intrathecal 2R, 6R-HNK observably reversed this increase. Immunofluorescent staining indicated that 42 μM 2R, 6R-HNK obviously suppressed the overexpression of CGRP in the bilateral DRGs and SDHs at 3 h (Fig. 6c, d) and 30 d after LF-PENS (Fig. 6e, f). Although LF-PENS caused similar early-and-long-term changes in microglia morphology and number as HFS stimulation model [6], intrathecal 2R, 6R-HNK did not reverse these alterations (Fig. 6g-i). Taken together, these data suggested that intrathecal 2R, 6R-HNK significantly inhibits neuronal hyperexcitability and CGRP upregulation.

### Intrathecal 2R,6R-HNK reverses the increase in TRPA1, TRPV1 and VGLUT2 expression and the co-expression with CGRP

**Fig. 7:**
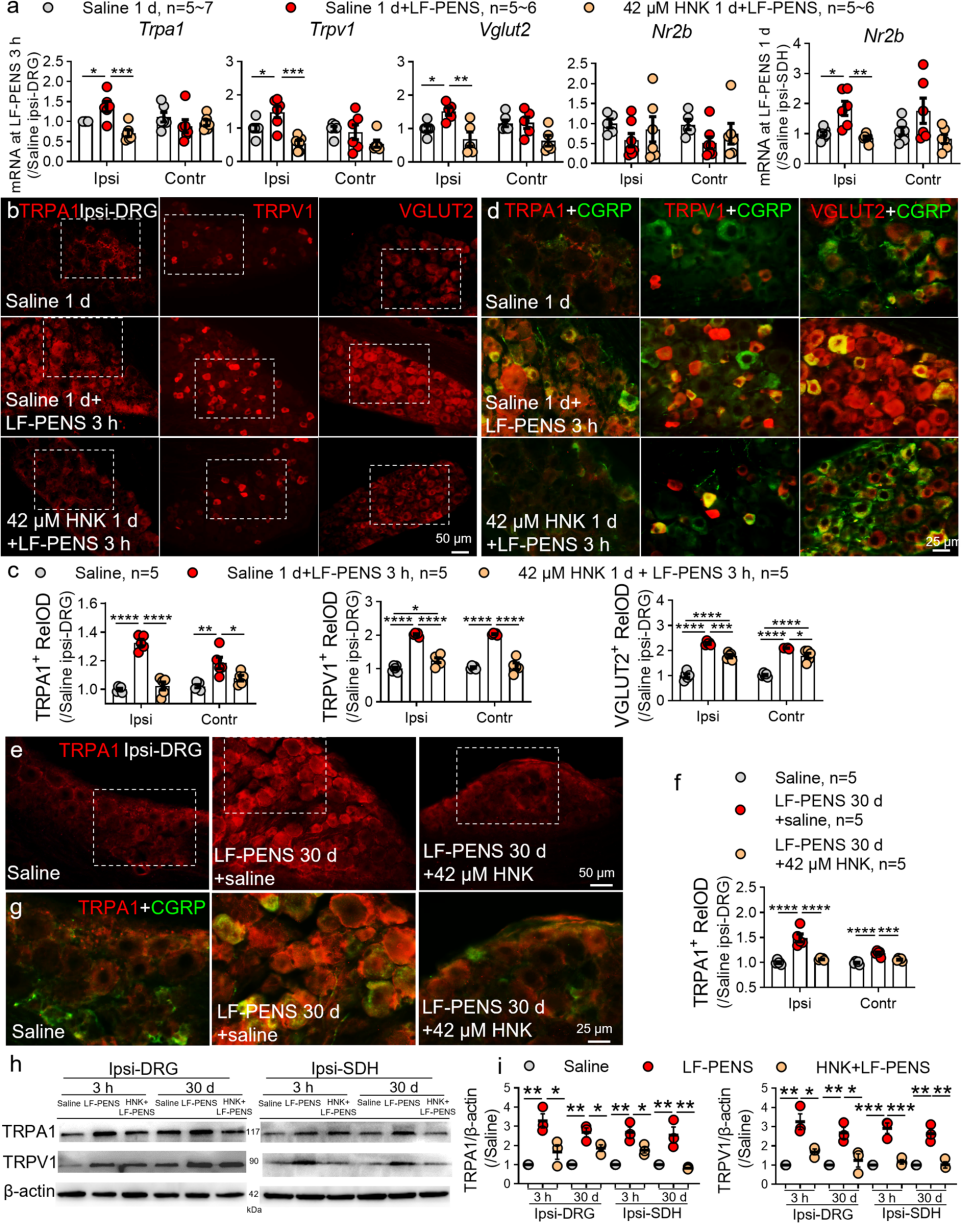
The increases in TRPA1/TRPV1 expression and their co-expression with CGRP are reduced by 2R, 6R-HNK. **a** RT-qPCR results showing the mRMA expressions mRNA in bilateral DRGs or SDH at 3 h/1 d after LF-PENS from different groups (*n* = 5 ~ 7 mice/group). **b-g** Representative photomicrographs of immunofluorescent (**b**, **e**), statistical analysis (**c**, **f**) and double-labeling staining (d, g, magnified from the white dotted boxed in the monochromatic micrographs b, e) and illustrated the changes of TRPA1, TRPV1 and the co-expression with CGRP in bilateral L4 DRGs at 3 h or 30 d after LF-PENS (*n* = 5 mice/group, 4 sections/mouse). **h**, **i** Western blot and statistical analysis showed the protein expressions in DRG and SDHs in each group (*n* = 3 mice/group). ^*^p < 0.05, ^**^p < 0.01, ^***^p < 0.001, ^****^p < 0.0001 between groups

Given the critical roles of TRPA1, TRPV1 and VGLUT2 in the development of pain and CGRP expression or releasing [58–60], we established the acute impact of 2R, 6R-HNK on these molecules in DRG neurons to explore the mechanism. Intrathecal 2R, 6R-HNK administration essentially blocked the increases of *Trpa1*, *Trpv1*, *Vglut2* mRNA in ipsilateral DRGs at 3 h after LF-PENS and *Nr2b* in ipsilateral SDH at 1 d but not 3 h (Fig. 7a). Immunofluorescence staining further demonstrated that TRPA1, TRPV1 and VGLUT2 were up-regulated in most cells of bilateral L4 DRG 3 h after LF-PENS (Fig. 7b-d), and TRPA1 progressively increased at 30 d (Fig. 7e-g). Notably, in addition to small-diameter DRG neurons, these pain-related molecules in model were also remarkably upregulated in medium and large ones. More importantly, these increased molecules were mostly co-stained with upregulated CGRP not only at 3 h after LF-PENS but also at 30 d (Fig. 7d, g), and 2R, 6R-HNK significantly reversed these pathological changes. Furthermore, Western blot results further validated changes in expression of TRPA1 and TRPV1 in the ipsilateral L4 DRGs and revealed similar changes in SDH (Fig. 7h, i). Thus, in parallel with the regulation of CGRP, 2R, 6R-HNK also inhibits the expressions of major TRPs and their functional partner VGLUT2.

### 2R, 6R-HNK preincubation suppresses Ca^2+^ response and CGRP expression evoked by TRPA1 or/and TRPV1 activation in cultured DRG neurons

**Fig. 8:**
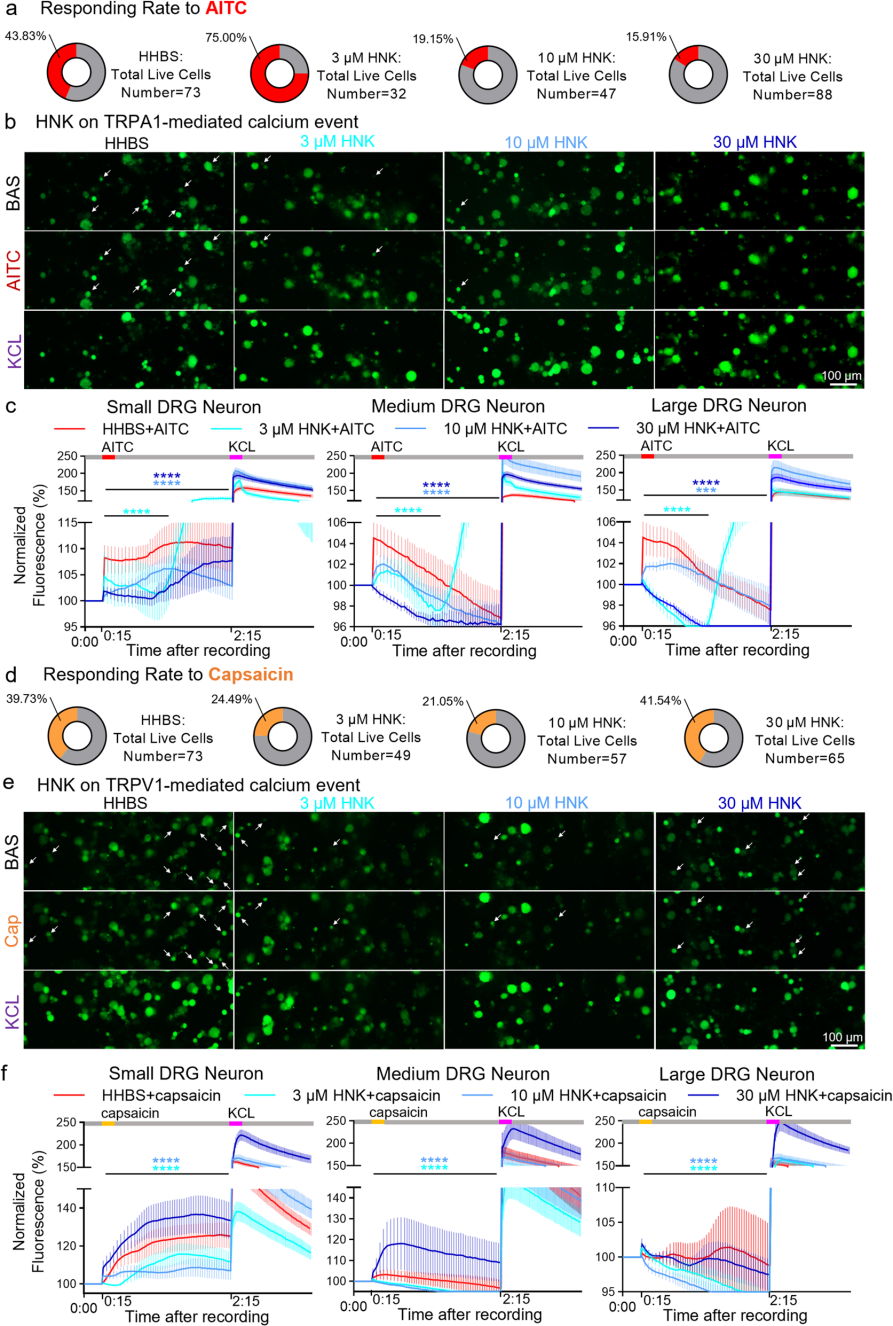
Pretreatment with 2R, 6R-HNK blockes TRPA1- or TRPV1- activated calcium influx in DRG neurons. **a** Statistical donut charts showed the percentage of responding neurons to allyl-isothiocyanate (AITC, an agonist of TRPA1 receptor) in different groups. **b** Representative images of Ca^2+^ responses in cultured DRG neurons before or during perfusion of AITC (100 μM, 120 s) and KCL (144 mM, 30 s) with HHBS or different dosage of 2R, 6R-HNK (3, 10, 30 μM) preincubation 3 h before trials. **c** Summary of Ca^2+^ responses in small (< 18 μm), medium (≥ 18 μm and ≤ 25 μm) and large (> 25 μm) DRG neurons pretreated with 2R, 6R-HNK or not. **d-f** Representative images (**e**), percentage of responding neurons (**d**) and time course plots (**f**) showing the changes in Ca^2+^ responses to capsaicin (Cap, TRPV1 agonist, 1 μM, 120 s) and KCL with or without 2R, 6R-HNK pretreatment, ^***^p < 0.001, ^****^p < 0.0001 compared with HHBS group

**Fig. 9:**
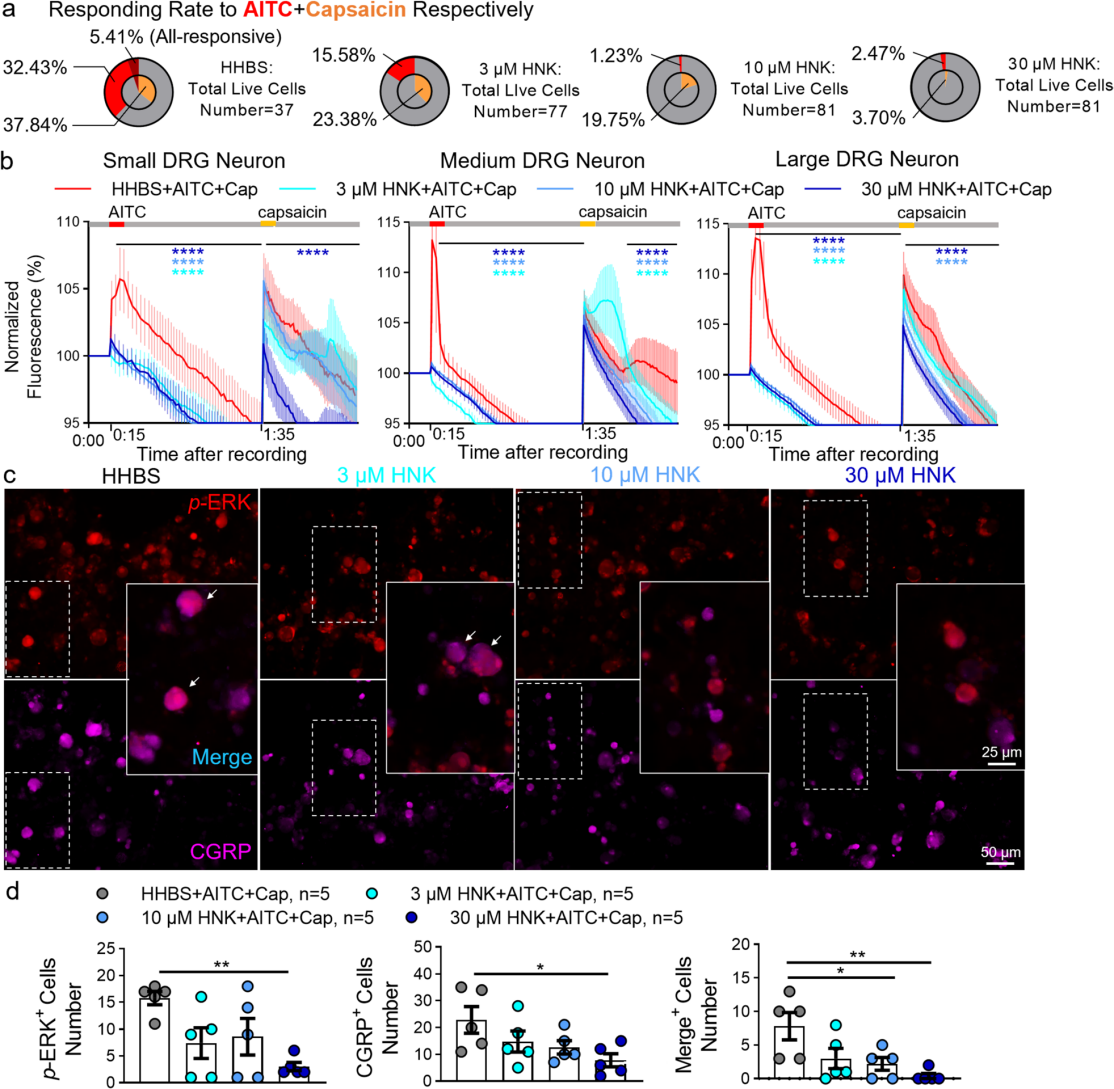
Ca^2+^ response and CGRP upregulation evoked by the activation of TRPA1 and TRPV1 are restrained by 2R, 6R-HNK. **a** Statistical donut charts indicated the effect of 2R, 6R-HNK pretreatment on the ratio of responding neurons to sequential stimuli of AITC-and-capsaicin with 80-s interval. **b** Statistics graphs of Ca^2+^ responses in small, medium and large DRG neurons with 2R, 6R-HNK preincubation or not, ^****^p < 0.0001 compared with HHBS group. **c-d** After calcium imaging, representative images of immunofluorescence (**c**) and cytometry (**d**) showed the changes of excitability and CGRP expression in DRG neurons after concomitant administration of AITC-and-capsaicin with or without 2R, 6R-HNK pretreatment (*n* = 5 samples/group), ^*^p < 0.05, ^**^p < 0.01 compared with each groups

We next performed in vitro calcium imaging to examine whether 2R, 6R-HNK impacted the activity of sensory neurons by affecting TRPA1/TRPV1 alone or together. Under vehicle conditions, 43.84% of live DRG neurons responded to 100 μM TRPA1 agonist AITC during 120-s trail (Fig. 8a). Interestingly, preincubation with 10 and 30 μM 2R, 6R-HNK effectively eliminated the proportion of responding cells to 19.15% and 15.91%, respectively. Of note, 2R, 6R-HNK blocked the AITC-evoked Ca^2+^ response in all sized DRG neurons with concentration dependence (Fig. 8b, c). In contrast, 3 μM 2R, 6R-HNK did not suppress the rate of total responded cells, but dramatically postponed AITC-induced calcium peak (Fig. 8a-c). In addition, 3 and 10 μM 2R, 6R-HNK dose-dependently abolished the evoked Ca^2+^ influxes induced by 1 μM TRPV1 agonist capsaicin mainly in small-diameter neurons (Fig. 8d-f). Unlike TRPA1, 30 μM 2R, 6R-HNK contradictorily facilitated TRPV1 activation by capsaicin. Given TRPA1 has a complex interaction and collaboration with TRPV1 [61], and TRPA1 increased juxtaposed with TRPV1 in our model, sequential stimuli with AITC and then capsaicin were set to observe reciprocal actions of TRPs. In control group, few cells (5.41%) responded to AITC and capsaicin because the 80-s interval is shorter than the time it took for neurons to fully regain their ability to respond (Fig. 9a). To our surprise, 2R, 6R-HNK reduced both the percentages of AITC- and Cap-responding neurons in an approximately dose-dependent manner (Fig. 9a, b). More importantly, 30 μM 2R, 6R-HNK not only stably blocked TRPA1 activation, but also promoted a cooperative inhibition on TRPV1 (Fig. 9a, b). Subsequent immunofluorescence results showed that 2R, 6R-HNK suppressed the expression and co-expression of *p*-ERK and CGRP in a concentration-dependent manner (Fig. 9c, d). These findings imply that 2R, 6R-HNK could synergistically affect the function of TRPA1 and TRPV1 channels to inhibit neuronal excitability and CGRP overexpression.

### The inhibitive effects of 2R, 6R-HNK on CGRP expression and CPP are occluded by blocking TRPA1

**Fig. 10:**
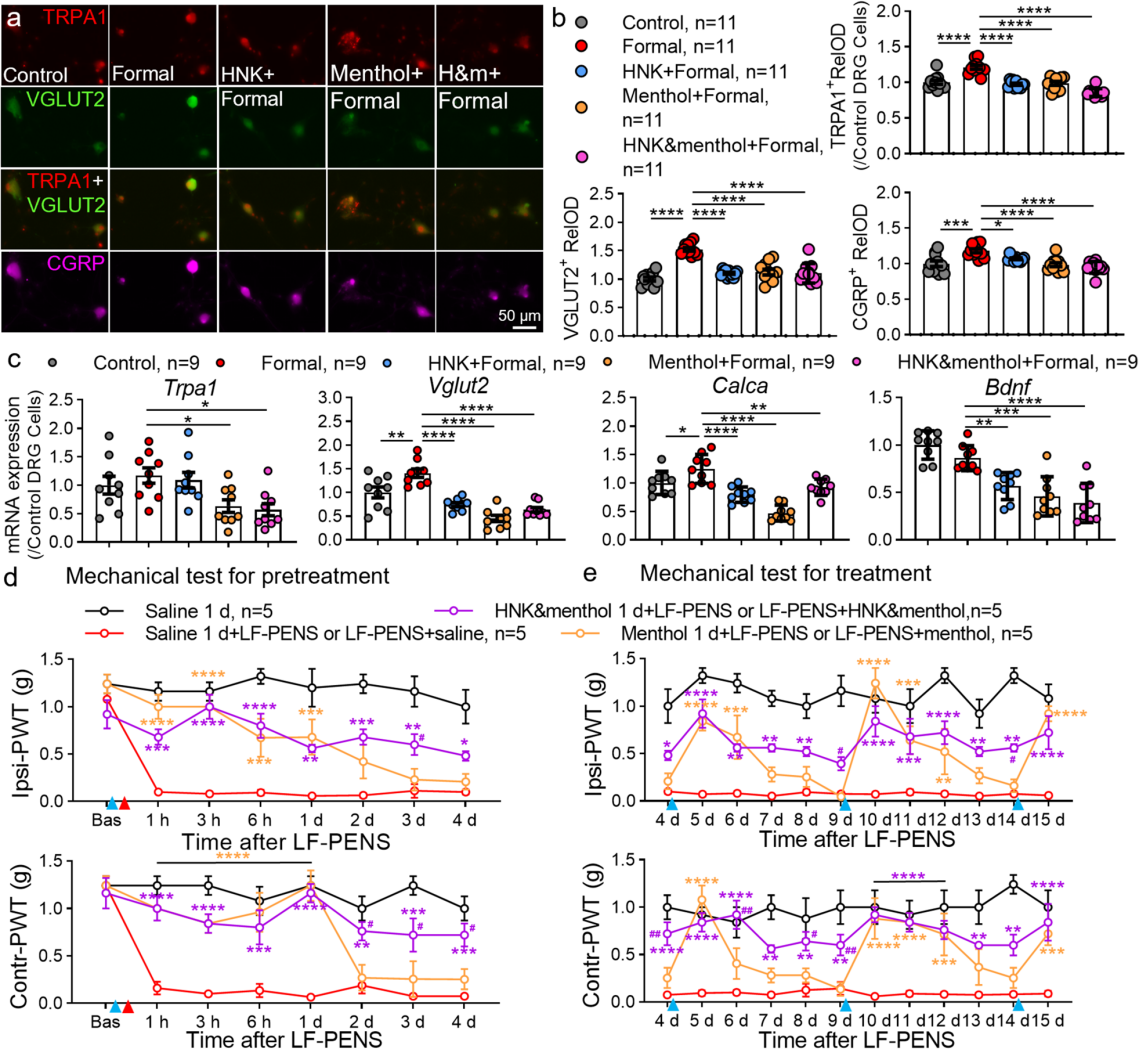
The inhibitory effects of 2R, 6R-HNK on CGRP expression and allodynia are occluded by TRPA1 inhibitor menthol. **a**, **b** Representative images and RelOD statistics show the changes of TRPA1, VGLUT2 and CGRP expression in DRG neurons 24 h after the TRPA1 agonist formaldehyde (Formal, 30 μM) incubation with or without 30 μM 2R, 6R-HNK and/or 300 μM menthol. Before that, DRG neurons were planted under equal density, and test started when cells reconstructed their synaptic growth at 48 h (*n* = 11 samples/group). **c** Quantification of *Trpa1*, *Vglut2*, *Bdnf* and *Calcα* mRNA in DRG neurons in vitro from different groups 24 h after formaldehyde stimulation (*n* = 9 samples /group), ^*^p < 0.05, ^**^p < 0.01,^***^p < 0.001, ^****^p < 0.0001 between groups. **d**, **e** The preventive (**d**) and therapeutic (**e**) effects of TRPA1 inhibition by menthol and 2R, 6R-HNK on LF-PENS-induced mechanical pain were mostly coupled (*n* = 5 mice/group), ^*^p < 0.05, ^**^p < 0.01, ^***^p < 0.001, ^****^p < 0.0001 compared with saline 1 d + LF-PENS or LF-PENS + saline group; ^#^*p* < 0.05 compared with Menthol 1 d + LF-PENS or LF-PENS + menthol group

To further address whether blocking TRPA1 is required for the inhibitory effects of 2R, 6R-HNK on CGRP expression and CPP, we used TRPA1 agonist formaldehyde or antagonist menthol [36, 62] as pharmacologic inhibition or activation. In vitro DRG cells culture experiments, capsazepine (a TRPV1 antagonist) was preincubated to avoid TRPV1 interference. Consistent with a previous study [36], 30 μM formaldehyde (a TRPA1 agonist) markedly increased the expression of TRPA1 protein at 24 h after treatment (Fig. 10a, b), indicating the function and expression of TRPA1 may have positive feedback. Formaldehyde also led to the upregulation in VGLUT2 and CGRP which were co-stained with TRPA1. Conversely, 30 μM 2R, 6R-HNK preincubation basically reversed the changes of TRPA1, VGLUT2 and CGRP. Blocking TRPA1 by menthol (300 μM) had a similar inhibitory effect on these three molecules and a combined occluding effect occurred when applied together. In addition, RT-qPCR analysis showed that only VGLUT2 and CGRP mRNA were increased after Formaldehyde treatment for 24 h (Fig. 10c). 2R, 6R-HNK pretreatment had no effect on TRPA1 mRNA expression, but decreased the increases of VGLUT2, CGRP and BDNF. Menthol alone or in combination with 2R, 6R-HNK clearly decreased all the molecules, suggesting that 2R, 6R-HNK exert its inhibitory effect by blocking TRPA1. To be mentioned, menthol exerted better inhibitive effect on increased CGRP mRNA than 2R, 6R-HNK (^**^p = 0.0043) or combination of the two (^****^p < 0.0001) after 1 d formaldehyde treatment. Finally, in LF-PENS-induced acute pain experiments, intrathecal treatment of menthol before or 4 d after stimulation significantly increased bilateral PWT from 1 h to 2 d, as well as the combination with 30 μM 2R, 6R-HNK (Fig. 10d). Interestingly, therapeutic administration of menthol in chronic pain reached its peak antinociceptive effect soon and dropped then, while multiple 2R, 6R-HNK guaranteed a comparatively stable analgesia (Fig. 10e). These data suggested that blocking TRPA1 might be a key mechanism by which intrathecal 2R, 6R-HNK exerts the therapeutic and prophylactic effects on CPP.

## Discussion

**Fig. 11:**
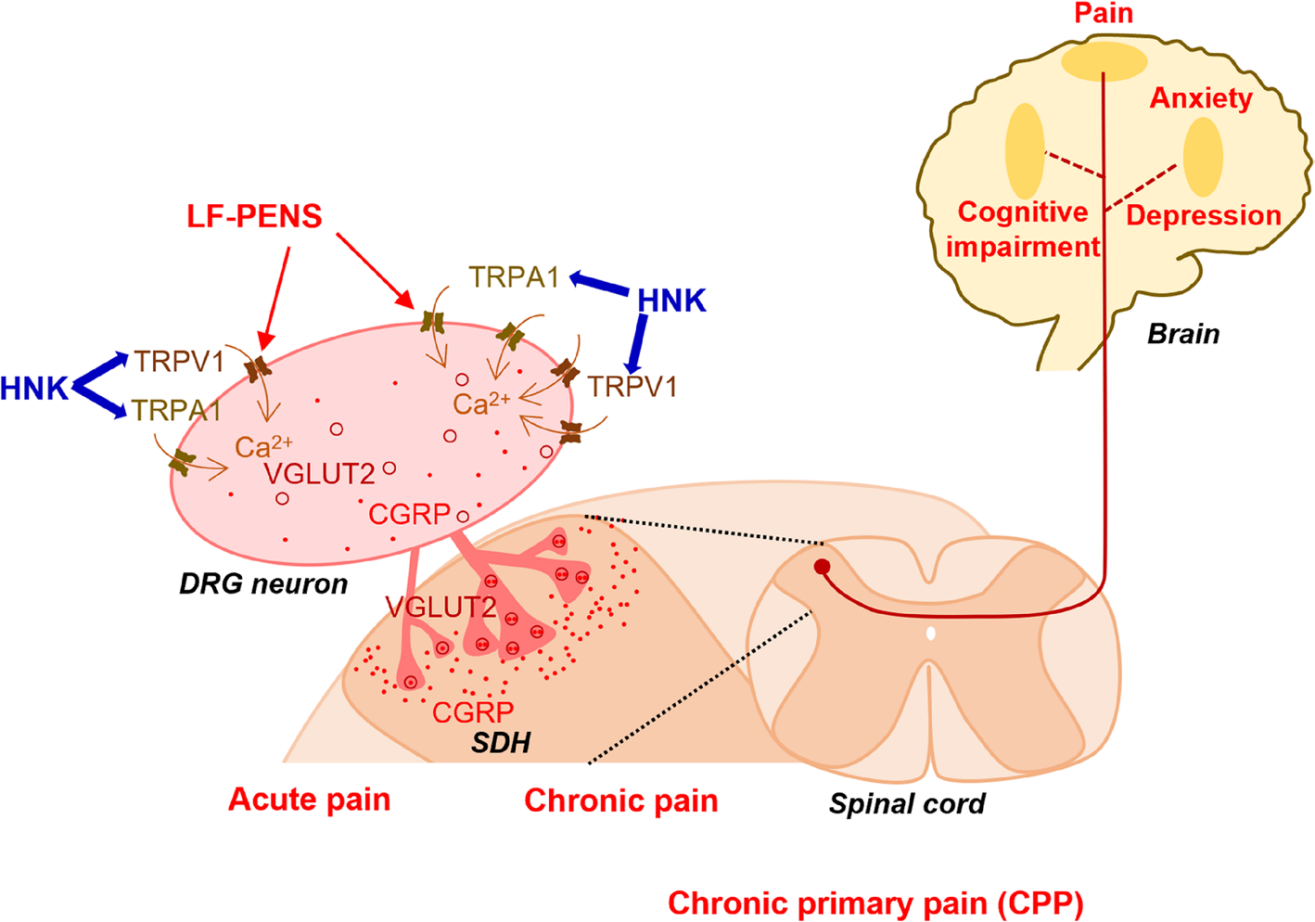
Schematic diagrams of intrathecal 2R, 6R-HNK analgesia mechanism of in LF-PENS-induced CPP mouse model. A novel mouse model of CPP with comorbidities (anxiety, depression and/or cognitive impairment) is established by noninvasive LF‑PENS of popliteal fossa. Intrathecal 2R, 6R‑HNK produces potent analgesia on LF‑PENS‑induced CPP and alleviates the comorbidities depression and cognitive impairment in dose‑dependent manner. Mechanically, 2R, 6R‑HNK not only suppresses the upregulation of TRPA1 and other functional partners (including CGRP, TRPV1 and VGLUT2) in DRG neurons and neuronal excitability in the ascending pain pathways, but also decreases TRPA1‑ and TRPV1‑mediated Ca2+ influxes and CGRP overexpression in cultured DRG neurons. Together, TRPA1 is critical for the occurrence and development of CPP, as well as the potent analgesia of intrathecal 2R, 6R‑HNK

CPP is a challenge in both preclinical pain research and clinical medicine due to the lack of suitable animal models and the complex and unclear mechanisms. In this study, we established a novel noninvasive animal model of CPP with the comorbidities of anxiety, depression and cognitive impairment by using LF-PENS that mimics abnormal neuronal discharges of the nociceptive nerves [63]. We found that intrathecal 2R, 6R-HNK produces long-lasting and intense analgesia in LF-PENS induced acute and CPP pain states as well as in HFS-induced nociplastic pain. Mechanistically, we identified that 2R, 6R-HNK mainly affected the expression and function of TRPA1 in DRG neurons, which resulted in the decreased expression of CGRP (Fig. 11).

### A newly created mouse model of CPP induce by 10 V LF-PENS

CPP is characterized by severe pain with no clear underlying cause, and exists different subclasses and high heterogeneity. However, patients with different CPP subtypes often experience sensitization of the nervous system due to changes in neurons [64]. Most animal models of different CPP subclasses were built upon enhancing neuronal excitability, e.g., trinitrobenzene sulfonic acid (TNBS)-modified potassium channel for BMS [65], stress-based (cold, sound, or swim) approaches for fibromyalgia [66] or IBS [67], and electrical, physical or chemical stimulation of the trigeminal ganglion for chronic migraine [68]. Accordingly, CPP has a common pathogenesis–nociplastic pain–only central sensitization without significant tissue damage [4]. It is known that central sensitization is essential in the pathogenesis of maintaining chronic pain and spinal LTP of C-fiber-evoked field potentials is considered as a synaptic model for central pain sensitization [69, 70]. For these reasons, CPP models can be created by using LTP-inducible electrical stimulation to simulate abnormal neural excitation without peripheral damage. Our previous study demonstrated that 10 V LTP-inducible HFS or LFS directly on sciatic nerve produced nociplastic pain without significant nerve injury [6], which was roughly consistent with the characteristics of CPP. However, this model presents skin and muscle injuries. By using a modified method of percutaneous electric nerve stimulation (PENS), we found that LF-PENS with the same parameters (10 V LFS) caused not only bilateral chronic mechanical and cold pain (Fig. 2), but also emotional and cognitive impairment (Fig. 3). These behavioral changes in this model are basically consistent with the characteristics of CPP. Unlike analgesic transcutaneous electrical nerve stimulation (TENS), which reduces the activity and excitability of central pain-transmitting neurons by activating non-noxious low threshold A-fibers [71, 72], LF-PENS used in our pain model in mouse is 10 V noninvasive voltage stimulation to excite the pain-conducting C-fibers. In addition to the peripheral pain pathways in DRGs and SDHs (Fig. 5), aberrant neuronal activity is observed in Amy, LHb, ACC and other brain regions that are associated with pain-related adverse emotion, cognition, memory, social function and possible autonomic nervous system changes (Fig. 4). Moreover, the important pain-related molecules such as CGRP and TRPs were significantly increased in our model (Figs. 6, 7). CGRP levels were particularly elevated in many clinical CPP subtypes and animal models (such as primary trigeminal neuralgia [73, 74], migraine [56], IBS [75] and fibromyalgia syndrome [76, 77], and inhibiting or blocking CGRP signaling pathway is an effective strategy to treat migraine [78, 79]. Besides, the overexpression of CGRP^+^ terminals in the superficial SDH was still increased at 30 d following LF-PENS (Fig. 6e, f). This suggested that LF-PENS induced CPP exhibited structural plasticity changes consistent with HFS-induced nociplastic pain, which is an important basis for LF-PENS-induced chronic pain. Up to now, we have considered that 10 V LF-PENS-induced chronic pain can be used as a common mouse model of CPP, especially for skeletal muscle pain and idiopathic low back pain.

Unilateral C-fiber-activated electrical stimulation caused bilateral pain and increased excitability of peripheral and central neurons may be related to heterogenous sensitization of pain transmission by heterosynaptic LTP [80–82] and glial LTP [83], but the mechanism still remains to be further investigated.

### LF-PENS-induced CPP is combined with affective and cognitive impairment

Accumulating evidence has shown that chronic pain is associated with comorbidities such as cognitive impairment and emotional disorders (anxiety or depression), as well as molecular, functional and structural changes in many brain regions [84, 85]. Various stressors (such as pain) and negative emotional stimuli may induce neuronal activation (indicated by the expressions of c-Fos, *p*-ERK, *p*-CREB or calcium activity) in these brain regions, such as LHb [41]. For example, Amy is a limbic region that plays a key role in emotional-affective behaviors (fear memory, anxiety-like behaviors and pain-induced aversion) and pain modulation [39], and enhanced c-Fos expression in the central Amy correlates with nociceptive input [86] or aversive stimuli [87]. A previous study suggests that in the early phase of postoperative pain, pain-related anxiety and mechanical hypersensitivity are tightly linked and regulated by the increase of *p*-ERK in the ACC, while in the late phase, ERK activation in the ACC is only required for the expression of pain-related anxiety [88]. Our IF results showed that the three molecular markers of neuronal activity (c-Fos, *p*-CREB, *p*-ERK) were increased in Amy, Pir, LHb, VMH and DM in the early phase (3 h after LF-PENS) and in ACC, Pir and VMH in the later phase (30 d after LF-PENS) (Fig. 4). And these changes are consistent with the behaviors of pain and its comorbidities. Based on these studies and our findings, we suggest that the changes of these compatible molecular markers (c-Fos, *p*-CREB, *p*-ERK) in different nuclei may correspond to the changes of chronic pain and its comorbidities in CPP. However, these issues remain to be further addressed.

### Intrathecal 2R, 6R-HNK is effective for CPP

Previous preclinical evidence has demonstrated that i.p. injection of 2R, 6R-HNK (10 mg·kg^−1^) had antinociceptive and analgesic effects in neuropathic pain [16], CRPS-1, postoperative pain [18] and inflammatory pain [16]. A recent study reported that single-dose intranasal 2R, 6R-HNK (10 mg·kg^−1^) rapidly improved thermal response in control mice, nociceptive response and anxiety levels during the second tonic phase of the formalin test [17]. To date, the analgesic effect of 2R, 6R-HNK remains elusive. To explore the possible targets of 2R, 6R-HNK, we first evaluated the i.p. and i.t. delivery systems of 2R, 6R-HNK on analgesic efficiency. Compared with i.p. injection of equal concentration, a single intrathecal 2R, 6R-HNK had stronger and longer antinociception at 3-w after HFS from 6 h to 4 d after administration (Fig. 1a). These results suggested that DRG and/or SDH may be the direct targets of 2R, 6R-HNK. We also investigated the analgesic efficiency of intrathecal S-Ketamine and 2R, 6R-HNK at the same dose and found that intrathecal 2R, 6R-HNK, but not S-Ketamine, delayed the occurrence of HFS-induced mechanical hypersensitivity (Fig. 1b). This is consistent with previous studies showing that i.p. injection of 2R, 6R-HNK produces a delayed antinociceptive effect while ketamine has a transient short-lived antinociception [16].

Subsequently, several behavioral results in the newly developed CPP model further established that multiple intrathecal 2R, 6R-HNK relieved bilateral mechanical, cold allodynia for several days in a dose-dependent manner (Fig. 2). Additionally, intrathecal pre-administration of 2R, 6R-HNK completely blocked acute mechanical pain and spontaneous pain-like behaviors induced by LF-PENS, and partially suppressed thermal pain (Fig. 5a-e). In brief, this study is the first to report that i.t. 2R, 6R-HNK displays an effective analgesic effect on CPP. Moreover, multiple intrathecal administration of 2R, 6R-HNK did not reduce the analgesia and produce tolerance, but rather produced an additive effect. In addition, intrathecal 2R, 6R-HNK also displayed an antidepressant effect in the CPP model (Fig. 3a-c), in line with a previous report [10] of i.p. administration. More rarely, multiple 2R, 6R-HNK reduced the comorbidity of memory impairment in CPP (Fig. 3d). Accordingly, our findings declare that intrathecal administration of 2R, 6R-HNK provide a satisfactory therapeutic effect for CPP.

In this study, intrathecal administration of 2R, 6R-HNK has a stronger and long-lasting analgesic effect, which is not only superior to the duration of i.p. or intranasal delivery reported in other studies, but also significantly exceeded the cycle of 2R, 6R-HNK enrichment in tissues and normal metabolism of drug enzymes through the liver. There may be the following mechanisms and reasons: 1) I.p. injection has the problems of tissue enrichment, liver and kidney metabolism, half-life and low efficiencies of crossing blood–brain barrier and blood-spinal barrier. 2) The route of i.t. injection has the advantages of direct action on the nervous system, relatively higher local concentration and low local metabolism. 3) Our in vivo and in vitro results confirmed that intrathecal injection of 2R, 6R-HNK significantly inhibits the expressions and functions of TRPA1 and TRPA1 channels, neuronal excitability in the ascending pain pathways, and important molecules that affect the plasticity of pain synapses, such as CGRP, BDNF and VGLUT2. 4) Furthermore, given that i.t. injection can directly enter the subarachnoid space, the analgesic targets of 2R, 6R-HNK may not only target the adjacent DRG and/or SDH, but also act on the pain-related circuits of the brain. Taken together, our findings suggest that the analgesic effects of intrathecal 2R, 6R-HNK may be related to the direct action on the structural and functional plasticity of different target sites in pain pathways, especially DRG. However, to fully elucidate the pharmacodynamic properties of 2R, 6R-HNK, it is necessary to clarify whether it is directly bind to TRPA1, the domain and the characteristics of its action.

### 2R, 6R-HNK suppresses neuronal excitability and plasticity in the ascending pain pathway

It is known that 2R, 6R-HNK plays an antidepressant role by increasing glutamate release and α-amino-3-hydroxy-5-methyl-4-isoxazole-propionic acid receptors (AMPAR) expression to increase AMPAR-dependent synaptic transmission [10, 89]. By using antagonists, a previous study has found that AMPARs but not opioid receptors are involved in the initiation mechanism of 2R, 6R-HNK delayed analgesia [15]. However, 2R, 6R-HNK was i.p. injected and did not reflect the precise analgesic mechanism of 2R, 6R-HNK. Thus, the key target sites and molecular mechanisms of 2R, 6R-HNK analgesia remain unclear.

Subcutaneous (2R, 6R; 2S, 6S)-HNK (10 and/or 30 mg·kg^−1^) does not possess antinociceptive properties [90], while a single intranasal 2R, 6R-HNK (10 mg·kg^−1^) presented a fast-acting analgesia, with maximum efficacy observed 30 min after administration [17]. Intranasal delivery is a noninvasive way to get drug into the brain bypassing the blood–brain and blood-cerebrospinal fluid barriers [91]. Together, the difference in therapeutic efficacy between the two routes of administration implicates that 2R, 6R-HNK may exert its analgesic effect by acting directly on the nervous system. Intrathecal drug delivery system (IDDS) is well-established as an effective therapeutic approach to patients with chronic non-malignant or malignant pain in targeted medical actions upon spinal cords and DRGs [92]. Here, we found the analgesic effect of intrathecal 2R, 6R-HNK was superior that of i.p. injection in both magnitude and duration (Fig. 1a), strongly suggesting that the key analgesic site(s) of intrathecal 2R, 6R-HNK may be related to the direct effect on DRG and/or SDH. This hypothesis was confirmed by the following series of experiments: 1) LF-PENS-induced bilateral mechanical allodynia, spontaneous pain and thermal hyperalgesia were rapidly alleviated by pre-intrathecal 2R, 6R-HNK in a dose–response (Fig. 5b-e); 2) Increased neuronal excitability (marked by *p*-ERK and c-Fos) in DRGs and SDHs (Fig. 5f-k) and upregulation of CGRP and BDNF at 3 h after LF-PENS were clearly suppressed by 2R, 6R-HNK (Figs. 6, 7); 3) Calcium influxes and the changes of pain-related molecules evoked by TRPV1 or/and TRPA1 agonists in cultured DRG neurons were restrained by 2R, 6R-HNK in an approximately dose-dependent manner (Figs. 8, 9). Therefore, we consider that DRG neurons may be the key sites for 2R, 6R-HNK analgesia. Although there is no direct electrophysiological evidence, based on the above findings, we suggest that 2R, 6R-HNK can inhibit neuronal excitability in the ascending pain pathway.

Previous studies have addressed that 2R, 6R-HNK produces longer-lasting antinociception than circulating drugs regardless of the type of pain [16]. However, the half-life for 2R, 6R-HNK is about 1 h in the mouse brain [10], whereas 0.2–0.8 h in the plasma [23]. Similarly, our results showed that a single intrathecal 2R, 6R-HNK suppressed nociplastic pain by LTP-inducing HFS (Fig. 1) and LF-PENS (Figs. 2, 5) for up to 4 days. Surprisingly, single or multiple intrathecal injections of 2R, 6R-HNK significantly inhibited acute or chronic enhancement of CGRP expression and CGRP terminal structural plasticity (Fig. 6b-f). Accordingly, the inhibition of long-term plasticity changes in the pain pathway may be responsible for the long-lasting sustained analgesia of 2R, 6R-HNK.

Our data showed that intrathecal 2R, 6R-HNK also clearly alleviated LF-PENS-induced comorbidities depression and cognitive deficits (Fig. 3), and neuronal hyperexcitability in related brain regions (Fig. 4). This may be related to the direct action of 2R, 6R-HNK in brain regions, or an inhibition of pain pathway, which is worthy of further study.

### 2R, 6R-HNK suppresses CGRP expression mainly by regulating TRPA1 channels

CGRP is highly expressed in trigeminal ganglion (TG) and DRG primary sensory neurons of various CPP, such as idiopathic trigeminal neuralgia [73], IBS, Fibromyalgia [77], and is a hallmark and clinical therapeutic target of migraine attack [79]. In the current study, we demonstrated that intrathecal 2R, 6R-HNK suppressed LF-PENS-induced acute or chronic CGRP overexpression in DRG neurons and the superficial SDH, but did not inhibit microglia activation (Fig. 6c-i). 2R, 6R-HNK also inhibited the overexpression and co-staining of CGRP and *p*-ERK (a neuronal excitability marker) in cultured DRG neurons (Fig. 9c, d). In short, 2R, 6R-HNK specifically leads to functional desensitization of CGRP-expressing DRG neurons and central synaptic terminals.

Preclinical and clinical evidence has highlighted the role of the activation of TRP channels (mainly TRPA1) in the pathophysiology of various chronic pain (such as migraine) by promoting CGRP release [93, 94]. Although TRPA1 can contribute to pain sensitivity on its own [95], it always has a complex interaction and collaboration with TRPV1 [61]. In recent years, it has also been found that the basis of their cooperation largely lays stress on TRPA1’s structural convenience of calcium modulation [96]. Since TRPA1 is the only TRPs member known to be sensitive to electrophiles [97], it should be directly activated by LF-PENS. As expected, TRPs channel (especially TRPA1) mRNA and protein were markedly upregulated by LF-PENS (Fig. 7). Amazingly, these changes were significantly abolished by intrathecal 2R, 6R-HNK. 2R, 6R-HNK also depressed TRPV1 or/and TRPA1 agonist-induced calcium influx into DRG neurons in a rough dose response (Figs. 8, 9). Additionally, 2R, 6R-HNK not only inhibited the increase in TRPA1 protein expression induced by TRPA1 agonist formaldehyde in cultured DRG neurons in vitro (Fig. 10a, b), but also delayed or attenuated the induction or maintenance of mechanical hypersensitivity induced by TRPA1 activation in vivo (Fig. 10d, e). Furthermore, the reversal effects of 2R, 6R-HNK on CGRP expression and pain were occluded by blocking TRPA1 by the antagonist menthol. It is worth mentioning that 2R, 6R-HNK also decreased the upregulation of VGLUT2 (Figs. 7a, 10c), a key mediator for glutamate transmission [98] and a major player in TRPV1 thermal nociception [99], and NR2B (a key subunit of NMDA receptor) in SDH. It has been reported that VGLUT2 mediates glutamatergic transmission in Trpv1-Cre afferents together with CGRP [100]. In cultured DRG neurons, 2R, 6R-HNK also reversed formaldehyde-induced increases of CGRP mRNA and protein, but its effect on mRNA was much weaker than menthol after 24 h incubation (Fig. 10a-c). Combined with the results of cell culture and behavior (Fig. 10), we considered that the effect of menthol is more rapid and transient, while that of 2R, 6R-HNK is relatively long-lasting. The effects of the two drugs were still chelated in general, but didn’t achieve completely consistent result in different time periods. Taken together, our findings suggest that 2R, 6R-HNK may suppress TRPA1 expression and function to affect TRPV1 channel, CGRP and VGLUT2, but further studies are needed.

## Conclusions

In summary, we have developed a distinctive mouse model of CPP with the comorbidities of anxiety, depression and cognitive impairment by using noninvasive LTP-inducible LF-PENS. On this basis, the antidepressant and analgesic effects of intrathecal 2R, 6R-HNK were first replicated in CPP, and the inhibitions of CGRP and TRPA1 upregulation in DRG neurons may be targets for the treatment of CPP. Our findings, to some extent, shed light on the key role of TRPA1 in the occurrence and development of CPP, as well as the promising prospect of intrathecal 2R, 6R-HNK in the therapeutic strategies against chronic pain.

### Supplementary Information

**Additional file 1:** **Table S1. **Summary of the statistical analyses.

## Data Availability

The data used and analyzed in this article are available upon reasonable request.

## Abbreviations

CPP: Chronic primary pain
2R, 6R-HNK: 2R, 6R-hydroxynorketamine
LTP: Long-term potentiation
HFS: High-frequency electrical stimulation
LFS: Low-frequency electrical stimulation
LF-PENS: Low-frequency percutaneous electrical nerve stimulation
i.p.: Intraperitoneally
i.t.: Intrathecally
RT-qPCR: Reverse transcription-quantitative real-time polymerase chain reaction
DRG: Dorsal root ganglion
CGRP: Calcitonin gene-related peptide
TRPA1: Transient receptor potential ankyrin 1
TRPV1: Transient receptor potential vanilloid-1
VGLUT2: Vesicular glutamate transporter-2
ICD-11: The 11th revision of the International Statistical Classification of Diseases and Related Health Problems
CRPS: Complex regional pain syndrome
BMS: Burning mouth syndrome
IBS: Irritable bowel syndrome
SDH: Spinal dorsal horn
NMDAR: N-methyl-D-aspartate receptor
i.v.: Intravenous
p.o.: Per os
PWT: Paw withdrawal threshold
Bas: Basal threshold
OFT: Open field test
SPT: Sucrose preference test
TST: Tail suspension test
FST: Forced swimming test
NOR: New-object recognition
EPM: Elevated plus maze test
IF: Immunofluorescence
PFA: Paraformaldehyde
RT: Room temperature
RelOD: Relative optical density
WB: Western blot
PLL: Poly-L-Lysine
AITC: Allyl-isothiocyanate
Cap: Capsaicin
Ipsi: Ipsilateral
Contr: Contralateral
Amy: Amygdala
Pir: Piriform cortex
LHb: Lateral habenular nucleus
ACC: Anterior cingulate cortex
VMH: Ventromedial nucleus of hypothalamus
DM: Dorsomedial hypothalamic nucleus
*p*-CREB: Phosphorylation of cAMP-response-element-binding proteins
*p*-ERK: Phosphorylation of extracellular signal-regulated protein kinases
Cq: Cycle quantification
BDNF: Brain-derived neurotrophic factor
TNBS: Trinitrobenzene sulfonic acid
PENS: Percutaneous electric nerve stimulation
TENS: Transcutaneous electrical nerve stimulation
AMPAR: α-Amino-3-hydroxy-5-methyl-4-isoxazole-propionic acid receptors
IDDS: Intrathecal drug delivery system
TG: Trigeminal ganglion

## References

[1] Treede RD, Rief W, Barke A (2019). Chronic pain as a symptom or a disease: the IASP Classification of Chronic Pain for the International Classification of Diseases (ICD-11). Pain.

[2] Nicholas M, Vlaeyen JWS, Rief W (2019). The IASP classification of chronic pain for ICD-11: chronic primary pain. Pain.

[3] Hausteiner-Wiehle C, Henningsen P (2022). Nociplastic pain is functional pain. Lancet.

[4] Fitzcharles MA, Cohen SP, Clauw DJ, Littlejohn G, Usui C, Hauser W (2021). Nociplastic pain: towards an understanding of prevalent pain conditions. Lancet.

[5] Nijs J, Lahousse A, Kapreli E et al (2021) Nociplastic Pain Criteria or Recognition of Central Sensitization? Pain Phenotyping in the Past, Present and Future. J Clin Med 10(15). 10.3390/jcm10153203 10.3390/jcm10153203 PMC8347369 34361986

[6] Zhou LJ, Peng J, Xu YN et al (2019) Microglia Are Indispensable for Synaptic Plasticity in the Spinal Dorsal Horn and Chronic Pain. Cell Rep 27(13):3844–3859 e3846. 10.1016/j.celrep.2019.05.087 10.1016/j.celrep.2019.05.087 PMC7060767 31242418

[7] Zanos P, Moaddel R, Morris PJ (2018). Ketamine and ketamine metabolite pharmacology: Insights into therapeutic mechanisms. Pharmacol Rev.

[8] Pham TH, Gardier AM (2019). Fast-acting antidepressant activity of ketamine: highlights on brain serotonin, glutamate, and GABA neurotransmission in preclinical studies. Pharmacol Ther.

[9] Autry AE, Adachi M, Nosyreva E (2011). NMDA receptor blockade at rest triggers rapid behavioural antidepressant responses. Nature.

[10] Zanos P, Moaddel R, Morris PJ (2016). NMDAR inhibition-independent antidepressant actions of ketamine metabolites. Nature.

[11] Zanos P, Gould TD (2018). Mechanisms of ketamine action as an antidepressant. Mol Psychiatry.

[12] Moaddel R, Abdrakhmanova G, Kozak J (2013). Sub-anesthetic concentrations of (R, S)-ketamine metabolites inhibit acetylcholine-evoked currents in alpha7 nicotinic acetylcholine receptors. Eur J Pharmacol.

[13] Highland JN, Zanos P, Riggs LM (2021). Hydroxynorketamines: Pharmacology and Potential Therapeutic Applications. Pharmacol Rev.

[14] Lumsden EW, Troppoli TA, Myers SJ (2019). Antidepressant-relevant concentrations of the ketamine metabolite (2R,6R)-hydroxynorketamine do not block NMDA receptor function. Proc Natl Acad Sci U S A.

[15] Yost JG, Wulf HA, Browne CA, Lucki I (2022). Antinociceptive and Analgesic Effects of (2R,6R)-Hydroxynorketamine. J Pharmacol Exp Ther.

[16] Yost JG, Browne CA, Lucki I (2022). (2R,6R)-hydroxynorketamine (HNK) reverses mechanical hypersensitivity in a model of localized inflammatory pain. Neuropharmacology.

[17] Goswami N, Aleem M, Manda K (2023). Intranasal (2R, 6R)-hydroxynorketamine for acute pain: Behavioural and neurophysiological safety analysis in mice. Clin Exp Pharmacol Physiol.

[18] Kroin JS, Das V, Moric M, Buvanendran A (2019). Efficacy of the ketamine metabolite (2R,6R)-hydroxynorketamine in mice models of pain. Reg Anesth Pain Med.

[19] Carrier N, Kabbaj M (2013). Sex differences in the antidepressant-like effects of ketamine. Neuropharmacology.

[20] Franceschelli A, Sens J, Herchick S, Thelen C, Pitychoutis PM (2015). Sex differences in the rapid and the sustained antidepressant-like effects of ketamine in stress-naive and "depressed" mice exposed to chronic mild stress. Neuroscience.

[21] Moaddel R, Sanghvi M, Dossou KS et al (2015) The distribution and clearance of (2S,6S)-hydroxynorketamine, an active ketamine metabolite, in Wistar rats. Pharmacol Res Perspect 3(4):e00157. 10.1002/prp2.157 10.1002/prp2.157 PMC4492732 26171236

[22] Fukumoto K, Toki H, Iijima M (2017). Antidepressant Potential of (R)-Ketamine in Rodent Models: Comparison with (S)-Ketamine. J Pharmacol Exp Ther.

[23] Zanos P, Highland JN, Liu X (2019). (R)-Ketamine exerts antidepressant actions partly via conversion to (2R,6R)-hydroxynorketamine, while causing adverse effects at sub-anaesthetic doses. Br J Pharmacol.

[24] Jonas R, Klusch A, Schmelz M, Petersen M, Carr RW (2015). Assessment of TTX-s and TTX-r Action Potential Conduction along Neurites of NGF and GDNF Cultured Porcine DRG Somata. PLoS One.

[25] Cevikbas F, Wang X, Akiyama T (2014). A sensory neuron-expressed IL-31 receptor mediates T helper cell-dependent itch: Involvement of TRPV1 and TRPA1. J Allergy Clin Immunol.

[26] Deuis JR, Dvorakova LS, Vetter I (2017). Methods used to evaluate pain behaviors in rodents. Front Mol Neurosci.

[27] Kremer M, Becker LJ, Barrot M, Yalcin I (2021). How to study anxiety and depression in rodent models of chronic pain?. Eur J Neurosci.

[28] Greco R, Demartini C, Zanaboni AM, Tassorelli C (2018). Chronic and intermittent administration of systemic nitroglycerin in the rat induces an increase in the gene expression of CGRP in central areas: potential contribution to pain processing. J Headache Pain.

[29] Irwin S, Houde RW, Bennett DR, Hendershot LC, Seevers MH (1951). The effects of morphine methadone and meperidine on some reflex responses of spinal animals to nociceptive stimulation. J Pharmacol Exp Ther.

[30] Colburn RW, Lubin ML, Stone DJ (2007). Attenuated cold sensitivity in TRPM8 null mice. Neuron.

[31] Tappe-Theodor A, Kuner R (2014). Studying ongoing and spontaneous pain in rodents–challenges and opportunities. Eur J Neurosci.

[32] Steru L, Chermat R, Thierry B, Simon P (1985). The tail suspension test: a new method for screening antidepressants in mice. Psychopharmacology.

[33] Gai BM, Bortolatto CF, Bruning CA (2014). Depression-related behavior and mechanical allodynia are blocked by 3-(4-fluorophenylselenyl)-2,5-diphenylselenophene in a mouse model of neuropathic pain induced by partial sciatic nerve ligation. Neuropharmacology.

[34] Leger M, Quiedeville A, Bouet V (2013). Object recognition test in mice. Nat Protoc.

[35] Narita M, Kaneko C, Miyoshi K (2006). Chronic pain induces anxiety with concomitant changes in opioidergic function in the amygdala. Neuropsychopharmacology.

[36] Wang XL, Cui LW, Liu Z (2019). Effects of TRPA1 activation and inhibition on TRPA1 and CGRP expression in dorsal root ganglion neurons. Neural Regen Res.

[37] Walker SM, Westin BD, Deumens R, Grafe M, Yaksh TL (2010). Effects of intrathecal ketamine in the neonatal rat: evaluation of apoptosis and long-term functional outcome. Anesthesiology.

[38] Truin M, Janssen SP, van Kleef M, Joosten EA (2011) Successful pain relief in non-responders to spinal cord stimulation: the combined use of ketamine and spinal cord stimulation. Eur J Pain 15(10):1049 e1041–1049. 10.1016/j.ejpain.2011.04.004 10.1016/j.ejpain.2011.04.004 21565537

[39] Neugebauer V, Mazzitelli M, Cragg B, Ji G, Navratilova E, Porreca F (2020). Amygdala, neuropeptides, and chronic pain-related affective behaviors. Neuropharmacology.

[40] Wang H, Li F, Zheng X (2022). Social defeat drives hyperexcitation of the piriform cortex to induce learning and memory impairment but not mood-related disorders in mice. Transl Psychiatry.

[41] Hu H, Cui Y, Yang Y (2020). Circuits and functions of the lateral habenula in health and in disease. Nat Rev Neurosci.

[42] Barthas F, Sellmeijer J, Hugel S, Waltisperger E, Barrot M, Yalcin I (2015). The anterior cingulate cortex is a critical hub for pain-induced depression. Biol Psychiatry.

[43] Bao AM, Swaab DF (2019). The human hypothalamus in mood disorders: The HPA axis in the center. IBRO Rep.

[44] Munglani R, Hunt SP (1995). Molecular biology of pain. Br J Anaesth.

[45] Carlezon WA, Duman RS, Nestler EJ (2005). The many faces of CREB. Trends Neurosci.

[46] Ji RR, Baba H, Brenner GJ, Woolf CJ (1999). Nociceptive-specific activation of ERK in spinal neurons contributes to pain hypersensitivity. Nat Neurosci.

[47] Zhuang ZY, Xu H, Clapham DE, Ji RR (2004). Phosphatidylinositol 3-kinase activates ERK in primary sensory neurons and mediates inflammatory heat hyperalgesia through TRPV1 sensitization. J Neurosci.

[48] Lotsch J, Oertel BG, Felden L (2020). Central encoding of the strength of intranasal chemosensory trigeminal stimuli in a human experimental pain setting. Hum Brain Mapp.

[49] May A, Schwedt TJ, Magis D, Pozo-Rosich P, Evers S, Wang SJ (2018). Cluster headache Nat Rev Dis Primers.

[50] Watanabe M, Kopruszinski CM, Moutal A (2022). Dysregulation of serum prolactin links the hypothalamus with female nociceptors to promote migraine. Brain.

[51] Cao H, Gao YJ, Ren WH (2009). Activation of extracellular signal-regulated kinase in the anterior cingulate cortex contributes to the induction and expression of affective pain. J Neurosci.

[52] Tolomeo S, Christmas D, Jentzsch I (2016). A causal role for the anterior mid-cingulate cortex in negative affect and cognitive control. Brain.

[53] Martenson ME, Cetas JS, Heinricher MM (2009). A possible neural basis for stress-induced hyperalgesia. Pain.

[54] Pinto-Ribeiro F, Amorim D, David-Pereira A (2013). Pronociception from the dorsomedial nucleus of the hypothalamus is mediated by the rostral ventromedial medulla in healthy controls but is absent in arthritic animals. Brain Res Bull.

[55] Li K, Zhou T, Liao L (2013). betaCaMKII in lateral habenula mediates core symptoms of depression. Science.

[56] Russo AF, Hay DL (2022). CGRP physiology, pharmacology, and therapeutic targets: Migraine and beyond. Physiol Rev.

[57] Parisien M, Samoshkin A, Tansley SN (2019). Genetic pathway analysis reveals a major role for extracellular matrix organization in inflammatory and neuropathic pain. Pain.

[58] Caterina MJ, Julius D (2001). The vanilloid receptor: a molecular gateway to the pain pathway. Annu Rev Neurosci.

[59] Patapoutian A, Tate S, Woolf CJ (2009). Transient receptor potential channels: targeting pain at the source. Nat Rev Drug Discov.

[60] Liu Y, Abdel Samad O, Zhang L (2010). VGLUT2-dependent glutamate release from nociceptors is required to sense pain and suppress itch. Neuron.

[61] Bautista DM, Jordt SE, Nikai T (2006). TRPA1 mediates the inflammatory actions of environmental irritants and proalgesic agents. Cell.

[62] Talavera K, Startek JB, Alvarez-Collazo J (2020). Mammalian Transient Receptor Potential TRPA1 Channels: From structure to disease. Physiol Rev.

[63] Ikeda H, Stark J, Fischer H (2006). Synaptic amplifier of inflammatory pain in the spinal dorsal horn. Science.

[64] Tao ZY, Wang PX, Wei SQ, Traub RJ, Li JF, Cao DY (2019). The role of descending pain modulation in chronic primary pain: potential application of drugs targeting serotonergic system. Neural Plast.

[65] Shinoda M, Takeda M, Honda K (2015). Involvement of peripheral artemin signaling in tongue pain: possible mechanism in burning mouth syndrome. Pain.

[66] Brum ES, Becker G, Fialho MFP, Oliveira SM (2022). Animal models of fibromyalgia: What is the best choice?. Pharmacol Ther.

[67] Ohlsson B (2021) Theories behind the effect of starch‑ and sucrose‑reduced diets on gastrointestinal symptoms in irritable bowel syndrome (Review). Mol Med Rep 24(4). 10.3892/mmr.2021.12372 10.3892/mmr.2021.12372 PMC8404103 34414452

[68] Chou TM, Chen SP (2018). Animal models of chronic migraine. Curr Pain Headache Rep.

[69] Ji RR, Kohno T, Moore KA, Woolf CJ (2003). Central sensitization and LTP: do pain and memory share similar mechanisms?. Trends Neurosci.

[70] Liu XG, Zhou LJ (2015). Long-term potentiation at spinal C-fiber synapses: a target for pathological pain. Curr Pharm Des.

[71] Johnson MI, Paley CA, Jones G, Mulvey MR, Wittkopf PG (2022). Efficacy and safety of transcutaneous electrical nerve stimulation (TENS) for acute and chronic pain in adults: a systematic review and meta-analysis of 381 studies (the meta-TENS study). BMJ Open.

[72] Walsh DM, Howe TE, Johnson MI,Sluka KA (2009) Transcutaneous electrical nerve stimulation for acute pain. Cochrane Database Syst Rev (2):CD006142. 10.1002/14651858.CD006142.pub2 10.1002/14651858.CD006142.pub2 19370629

[73] Cornelison LE, Hawkins JL, Durham PL (2016). Elevated levels of calcitonin gene-related peptide in upper spinal cord promotes sensitization of primary trigeminal nociceptive neurons. Neuroscience.

[74] Qin ZL, Yang LQ, Li N (2016). Clinical study of cerebrospinal fluid neuropeptides in patients with primary trigeminal neuralgia. Clin Neurol Neurosurg.

[75] Ailani J, Kaiser EA, Mathew PG (2022). Role of Calcitonin Gene-Related Peptide on the Gastrointestinal Symptoms of Migraine-Clinical Considerations: A Narrative Review. Neurology.

[76] Noor-Mohammadi E, Ligon CO, Mackenzie K, Stratton J, Shnider S, Greenwood-Van Meerveld B (2021). A Monoclonal anti-calcitonin gene-related peptide antibody decreases stress-induced colonic hypersensitivity. J Pharmacol Exp Ther.

[77] Korucu RU, Karadag A, Tas A, Ozmen E, Hayta E,Silig Y (2020) Serum Calcitonin Gene-Related Peptide and Receptor Protein Levels in Patients With Fibromyalgia Syndrome: A Cross-Sectional Study. Arch Rheumatol 35(4):463–467. 10.46497/ArchRheumatol.2020.7783 10.46497/ArchRheumatol.2020.7783 PMC7945696 33758802

[78] Cernuda-Morollon E, Larrosa D, Ramon C, Vega J, Martinez-Camblor P, Pascual J (2013). Interictal increase of CGRP levels in peripheral blood as a biomarker for chronic migraine. Neurology.

[79] Dodick DW, Lipton RB, Ailani J (2019). Ubrogepant for the Treatment of Migraine. N Engl J Med.

[80] Fenselau H, Heinke B, Sandkuhler J (2011). Heterosynaptic long-term potentiation at GABAergic synapses of spinal lamina I neurons. J Neurosci.

[81] Chen QY, Chen T, Zhou LJ, Liu XG,Zhuo M (2018) [EXPRESS] Heterosynaptic LTP from the anterior cingulate cortex to the spinal cord in adult rats. Mol Pain:1744806918798406. 10.1177/1744806918798406 10.1177/1744806918798406 PMC6311562 30105926

[82] Klein T, Stahn S, Magerl W, Treede RD (2008). The role of heterosynaptic facilitation in long-term potentiation (LTP) of human pain sensation. Pain.

[83] Kronschlager MT, Drdla-Schutting R, Gassner M, Honsek SD, Teuchmann HL, Sandkuhler J (2016). Gliogenic LTP spreads widely in nociceptive pathways. Science.

[84] Kuner R, Kuner T (2021). Cellular Circuits in the Brain and Their Modulation in Acute and Chronic Pain. Physiol Rev.

[85] Kupers R, Kehlet H (2006). Brain imaging of clinical pain states: a critical review and strategies for future studies. Lancet Neurol.

[86] Morland RH, Novejarque A, Spicer C, Pheby T, Rice AS (2016). Enhanced c-Fos expression in the central amygdala correlates with increased thigmotaxis in rats with peripheral nerve injury. Eur J Pain.

[87] Shackman AJ, Fox AS (2016). Contributions of the Central Extended Amygdala to Fear and Anxiety. J Neurosci.

[88] Dai RP, Li CQ, Zhang JW (2011). Biphasic activation of extracellular signal-regulated kinase in anterior cingulate cortex distinctly regulates the development of pain-related anxiety and mechanical hypersensitivity in rats after incision. Anesthesiology.

[89] Pham TH, Defaix C, Xu X (2018). Common Neurotransmission Recruited in (R, S)-Ketamine and (2R,6R)-Hydroxynorketamine-Induced Sustained Antidepressant-like Effects. Biol Psychiatry.

[90] Lilius TO, Viisanen H, Jokinen V, Niemi M, Kalso EA, Rauhala PV (2018). Interactions of (2S,6S;2R,6R)-Hydroxynorketamine, a Secondary Metabolite of (R, S)-Ketamine, with Morphine. Basic Clin Pharmacol Toxicol.

[91] Laffleur F,Bauer B (2021) Progress in nasal drug delivery systems. Int J Pharm 607:120994. 10.1016/j.ijpharm.2021.120994 10.1016/j.ijpharm.2021.120994 34390810

[92] De Andres J, Hayek S, Perruchoud C et al (2022) Intrathecal Drug Delivery: Advances and Applications in the Management of Chronic Pain Patient. Front Pain Res (Lausanne) 3:900566. 10.3389/fpain.2022.900566 10.3389/fpain.2022.900566 PMC9246706 35782225

[93] Shatillo A, Koroleva K, Giniatullina R (2013). Cortical spreading depression induces oxidative stress in the trigeminal nociceptive system. Neuroscience.

[94] Spekker E, Kortesi T,Vecsei L (2022) TRP Channels: Recent Development in Translational Research and Potential Therapeutic Targets in Migraine. Int J Mol Sci 24(1). 10.3390/ijms24010700 10.3390/ijms24010700 PMC9820749 36614146

[95] Lin King JV, Emrick JJ, Kelly MJS et al (2019) A Cell-Penetrating Scorpion Toxin Enables Mode-Specific Modulation of TRPA1 and Pain. Cell 178(6):1362–1374 e1316. 10.1016/j.cell.2019.07.014 10.1016/j.cell.2019.07.014 PMC6731142 31447178

[96] Zhao J, Lin King JV, Paulsen CE, Cheng Y, Julius D (2020). Irritant-evoked activation and calcium modulation of the TRPA1 receptor. Nature.

[97] Suo Y, Wang Z, Zubcevic L et al (2020) Structural Insights into Electrophile Irritant Sensing by the Human TRPA1 Channel. Neuron 105(5):882–894 e885. 10.1016/j.neuron.2019.11.023 10.1016/j.neuron.2019.11.023 PMC7205012 31866091

[98] Rogoz K, Lagerstrom MC, Dufour S, Kullander K (2012). VGLUT2-dependent glutamatergic transmission in primary afferents is required for intact nociception in both acute and persistent pain modalities. Pain.

[99] Lagerstrom MC, Rogoz K, Abrahamsen B (2010). VGLUT2-dependent sensory neurons in the TRPV1 population regulate pain and itch. Neuron.

[100] Rogoz K, Andersen HH, Lagerstrom MC, Kullander K (2014). Multimodal use of calcitonin gene-related peptide and substance P in itch and acute pain uncovered by the elimination of vesicular glutamate transporter 2 from transient receptor potential cation channel subfamily V member 1 neurons. J Neurosci.

