# Supplementary material for: The potent analgesia of intrathecal 2R, 6R-HNK via TRPA1 inhibition in LF-PENS-induced chronic primary pain model
Source: J Headache Pain. 2023 Oct 19;24(1):141. doi: 10.1186/s10194-023-01667-1 (PMC10585932; doi:10.1186/s10194-023-01667-1)
Supplement: Supplementary file 1 — Additional file 1: Table S1. Summary of the statistical analyses. [file 10194_2023_1667_MOESM1_ESM.docx]

**Table S1. Summary of the statistical analyses**

| Figure | Sample size | Statistical methods | F/t/*P* value | Post hoc test | Significance |
| --- | --- | --- | --- | --- | --- |
| 1**a** | n=5 | Two-way ANOVA | - | Turkey’ multiple  comparison | - |
| 1**b** | n=5 | Two-way ANOVA | - | Turkey’ multiple  comparison | - |
| 2**d** | n=5,6 | One-way ANOVA | F(2,14)=0.06932, P=0.9333 | Turkey’ multiple  comparison | ns |
| 2**e** | n=5-8 | Two-way ANOVA | - | Turkey’ multiple  comparison | - |
| 2**f** | n=5-8 | Two-way ANOVA | - | Turkey’ multiple  comparison | - |
| 3**a**  1 w 2% sucrose and water consumption | n=12,14 | One-way ANOVA  One-way ANOVA | **2% surcose:**  F(3,48)=2.527, P=0.0685  **water:**  F(3,48)=0.1298, P=0.9419 | Turkey’ multiple  Comparison  Turkey’ multiple  comparison | ns  ns |
| 3**a**  3 w 2% sucrose and water consumption | n=12,14 | One-way ANOVA  Kruskal-Wallis test | **2% surcose:**  F(3,48)=1.183, P=0.3260  **water:**  Kruskal-Wallis statistic=13.25, P= 0.0041 | Turkey’ multiple  Comparison  Dunn's multiple comparison | ns  Saline vs. LF-PENS, P= 0.0156;  LF-PENS vs. LF-PENS+42 μM HNK, P= 0.0060; |
| 3**a**  1 w sucrose preference (%) | n=12,14 | One-way ANOVA | F(3,48)=0.3754, P=0.7711 | Turkey’ multiple  comparison | ns |
| 3**a**  3 w sucrose preference (%) | n=12,14 | Kruskal-Wallis test | Kruskal-Wallis statistic=13.40, P= 0.0038 | Dunn's multiple comparison | Saline vs. LF-PENS, P= 0.0375;  LF-PENS vs. LF-PENS+21 μM HNK, P= 0.0285;  LF-PENS vs. LF-PENS+42 μM HNK, P= 0.0046; |
| 3**b** | n=12,14 | One-way ANOVA | F(3,48)=8.432, P=0.0001 | Turkey’ multiple  comparison | Saline vs. LF-PENS, P= 0.0015;  LF-PENS vs. LF-PENS+21 μM HNK, P= 0.0013;  LF-PENS vs. LF-PENS+42 μM HNK, P= 0.0003; |
| 3**c** | n=12,14 | One-way ANOVA | F(3,48)=7.013, P=0.0005 | Turkey’ multiple  comparison | Saline vs. LF-PENS, P= 0.0028;  LF-PENS vs. LF-PENS+21 μM HNK, P= 0.0075;  LF-PENS vs. LF-PENS+42 μM HNK, P= 0.0009; |
| 3**d**  Exposure time | n=12,14 | One-way ANOVA  One-way ANOVA | **Old object:**  F(3,48)=1.082, P=0.3657  **New object:**  F(3,48)=1.436, P=0.2440 | Turkey’ multiple  Comparison  Turkey’ multiple  comparison | ns  ns |
| 3**d**  NOR index | n=12,14 | One-way ANOVA | F(3,48)=7.370, P=0.0004 | Turkey’ multiple  Comparison | Saline vs. LF-PENS, P= 0.0007;  LF-PENS vs. LF-PENS+21 μM HNK, P= 0.0103;  LF-PENS vs. LF-PENS+42 μM HNK, P= 0.0013; |
| 3**e**  Enter times | n=12,14 | One-way ANOVA  One-way ANOVA | **Open arm:**  F(3,48)=0.9212, P=0.4378  **Closed arm:**  F(3,48)=2.970, P=0.0410 | Turkey’ multiple  Comparison  Turkey’ multiple  comparison | ns  Saline vs. LF-PENS, P= 0.0439; |
| 3**e**  Open arm rate | n=12,14 | One-way ANOVA | F(3,48)=6.364, P=0.0010 | Turkey’ multiple  comparison | Saline vs. LF-PENS, P= 0.0006; |
| 4**c**  c-Fos in Amy | n=5 | Kruskal-Wallis test  One-way ANOVA | **Ipsi:**  Kruskal-Wallis statistic=9.909, P=0.0013  **Contr:**  F(2,12)=58.00, P<0.0001 | Dunn's multiple comparison  Turkey’ multiple  comparison | Saline vs. LF-PENS, *P=* 0.0106;  LF-PENS vs. HNK+ LF-PENS, *P=* 0.0389;  Saline vs. LF-PENS, P <0.0001;  Saline vs. HNK+LF-PENS, *P=* 0.0007;  LF-PENS vs. HNK+ LF-PENS, *P=* 0.0003; |
| 4**c**  c-Fos in Pir | n=5 | One-way ANOVA  One-way ANOVA | **Ipsi:**  F(2,12)=45.08, P<0.0001  **Contr:**  F (2, 12) = 69.89, P<0.0001 | Turkey’ multiple  comparison  Turkey’ multiple  comparison | Saline vs. LF-PENS, *P* <0.0001;  LF-PENS vs. HNK+ LF-PENS, *P* <0.0001;  Saline vs. LF-PENS, *P* <0.0001;  LF-PENS vs. HNK+ LF-PENS, *P* <0.0001; |
| 4**c**  p-CREB in LHb | n=5 | One-way ANOVA  One-way ANOVA | **Ipsi:**  F(2,12)=12.73, P=0.0011  **Contr:**  F(2,12)=19.85, P=0.0002 | Turkey’ multiple  comparison  Turkey’ multiple  comparison | Saline vs. LF-PENS, *P* =0.0011;  Saline vs. HNK+ LF-PENS, *P* =0.0076;  Saline vs. LF-PENS, *P* =0.0001;  Saline vs. HNK+LF-PENS, *P=* 0.0096;  LF-PENS vs. HNK+ LF-PENS, *P* = 0.0483; |
| 4**c**  p-ERK in VMH | n=5 | One-way ANOVA  One-way ANOVA | **Ipsi:**  F(2,12)=161.0, P<0.0001  **Contr:**  F(2,12)=121.9, P<0.0001 | Turkey’ multiple  comparison  Turkey’ multiple  comparison | Saline vs. LF-PENS, *P* <0.0001;  Saline vs. HNK+LF-PENS, *P* <0.0001;  LF-PENS vs. HNK+ LF-PENS, *P* <0.0001;  Saline vs. LF-PENS, *P* <0.0001;  Saline vs. HNK+LF-PENS, *P* <0.0001;  LF-PENS vs. HNK+ LF-PENS, *P* <0.0001; |
| 4**c**  p-ERK in DM | n=5 | Kruskal-Wallis test  One-way ANOVA | **Ipsi:**  Kruskal-Wallis statistic=10.09, P=0.0009  **Contr:**  F(2,12)=457.0, P<0.0001 | Dunn's multiple comparison  Turkey’ multiple  comparison | Saline vs. LF-PENS, *P* =0.0083;  LF-PENS vs. HNK+ LF-PENS, *P* =0.0470;  Saline vs. LF-PENS, *P* <0.0001;  Saline vs. HNK+LF-PENS, *P* = 0.0008;  LF-PENS vs. HNK+ LF-PENS, *P* <0.0001; |
| 4**e**  p-ERK in ACC | n=5 | Kruskal-Wallis test  One-way ANOVA | **Ipsi:**  Kruskal-Wallis statistic=10.74, P=0.0007  **Contr:**  F(2,12)=91.09, P<0.0001 | Dunn's multiple comparison  Turkey’ multiple  comparison | Saline vs. LF-PENS, *P* =0.0052;  LF-PENS vs. LF-PENS+HNK, *P* =0.0497;  Saline vs. LF-PENS, *P* <0.0001;  LF-PENS vs. HNK+ LF-PENS, *P* <0.0001; |
| 4**e**  p-ERK in Pir | n=5 | One-way ANOVA  One-way ANOVA | **Ipsi:**  F(2,12)=235.4, P<0.0001  **Contr:**  F(2,12)=151.9, P<0.0001 | Turkey’ multiple  comparison  Turkey’ multiple  comparison | Saline vs. LF-PENS, *P* <0.0001;  Saline vs. LF-PENS +HNK, *P* <0.0001;  LF-PENS vs. LF-PENS +HNK, *P* <0.0001;  Saline vs. LF-PENS, *P* <0.0001;  Saline vs. LF-PENS +HNK, *P* = 0.0001;  LF-PENS vs. LF-PENS +HNK, *P* <0.0001; |
| 4**e**  p-ERK in VMH | n=5 | Kruskal-Wallis test  Kruskal-Wallis test | **Ipsi:**  Kruskal-Wallis statistic=9.831, P=0.0011  **Contr:**  Kruskal-Wallis statistic=12.57, P<0.0001 | Dunn's multiple comparison  Dunn's multiple comparison | Saline vs. LF-PENS, *P* =0.0101;  LF-PENS vs. LF-PENS+HNK, *P* =0.0453;  Saline vs. LF-PENS, *P* =0.0012; |
| 5**b**  Mechanical test | n=5-7 | Two-way ANOVA | - | Turkey’ multiple  comparison | - |
| 5**c**  Spontaneous pain-like behaviors | n=5-7 | Two-way ANOVA | - | Turkey’ multiple  comparison | - |
| 5**d**  Hargreaves test | n=5-7 | Two-way ANOVA | - | Turkey’ multiple  comparison | - |
| 5**g**  p-ERK in DRG | n=5 | One-way ANOVA  One-way ANOVA | **Ipsi:**  F(2,12)=34.17, P<0.0001  **Contr:**  F(2,12)=14.80, P=0.0006 | Turkey’ multiple  comparison  Turkey’ multiple  comparison | Saline vs. LF-PENS, *P* <0.0001;  LF-PENS vs. LF-PENS +HNK, *P* <0.0001;  Saline vs. LF-PENS, *P* = 0.0010;  LF-PENS vs. LF-PENS +HNK, *P* = 0.0020; |
| 5**g**  c-Fos in DRG | n=5 | One-way ANOVA  One-way ANOVA | **Ipsi:**  F(2,12)=7.285, P=0.0085  **Contr:**  F(2,12)=1.467, P=0.2691 | Turkey’ multiple  comparison  Turkey’ multiple  comparison | Saline vs. LF-PENS, *P* =0.0144;  LF-PENS vs. LF-PENS +HNK, *P* =0.0180;  ns |
| 5**i**  p-ERK in SDH | n=5 | One-way ANOVA  One-way ANOVA | **Ipsi:**  F(2,12)=34.27, P<0.0001  **Contr:**  F(2,12)=21.84, P=0.0001 | Turkey’ multiple  comparison  Turkey’ multiple  comparison | Saline vs. LF-PENS, *P* <0.0001;  LF-PENS vs. LF-PENS +HNK, *P* <0.0001;  Saline vs. LF-PENS, *P* =0.0002;  LF-PENS vs. LF-PENS +HNK, *P* =0.0003; |
| 5**i**  c-Fos in SDH | n=5 | One-way ANOVA  One-way ANOVA | **Ipsi:**  F (2, 12) = 11.57, P=0.0016  **Contr:**  F(2,12)=0.5461, P=0.5929 | Turkey’ multiple  comparison  Turkey’ multiple  comparison | Saline vs. LF-PENS, *P* =0.0016;  LF-PENS vs. LF-PENS +HNK, *P* =0.0116;  ns |
| 5**k** | n=3 | One-way ANOVA  One-way ANOVA  One-way ANOVA  One-way ANOVA | **3 h DRG:**  F(2,6)=31.04, P=0.0007  **3 h SDH:** F(2,6)=13.89, P=0.0056  **30 d DRG:**  F(2,6)=18.44, P=0.0027  **30 d SDH:** F(2,6)=20.19, P=0.0022 | Turkey’s multiple  Comparison  Turkey’s multiple  Comparison  Turkey’s multiple  Comparison  Turkey’s multiple  comparison | Saline vs. LF-PENS, *P=*0.0064;  LF-PENS vs. HNK+ LF-PENS, *P=* 0.0006;  Saline vs. LF-PENS, *P=*0.0166;  LF-PENS vs. HNK+ LF-PENS, *P*=0.0061;  Saline vs. LF-PENS, *P=*0.0454;  LF-PENS vs. HNK+ LF-PENS, *P*< 0.0022;  Saline vs. LF-PENS, *P=*0.0174;  LF-PENS vs. HNK+ LF-PENS, *P*=0.0018; |
| 6**a** | n=6,8 | Unpaired t test  Unpaired t test | **Ipsi:**  F test:  P= 0.7611  t=2.881, df=12  **Contr:**  F test:  P= 0.8942  t=2.378, df=12 | - | P=0.0138  P=0.0349 |
| 6**b**  Calca | n=6,7 | One-way ANOVA  Kruskal-Wallis test | **Ipsi:**  F(2,13)=12.24, P=0.0010  **Contr:**  Kruskal-Wallis statistic=0.1103, P= 0.9511 | Turkey’ multiple  comparison  Dunn's multiple comparison | Saline vs. LF-PENS, *P* = 0.0039;  LF-PENS vs. HNK+LF-PENS, *P* = 0.0020;  ns |
| 6**b**  Calcb | n=5 | One-way ANOVA  One-way ANOVA | **Ipsi:**  F(2, 12)=5.634, P=0.0188  **Contr:**  F(2,12)=3.600, P=0.0596 | Turkey’ multiple  comparison  Turkey’ multiple  comparison | LF-PENS vs. HNK+LF-PENS, *P* = 0.0217;  ns |
| 6**b**  Bdnf | n=5,6 | Kruskal-Wallis test  One-way ANOVA | **Ipsi:**  Kruskal-Wallis statistic=8.587, P= 0.0075  **Contr:**  F(2,16)=1.116, P=0.3518 | Dunn's multiple comparison  Turkey’ multiple  comparison | LF-PENS vs. HNK+LF-PENS, *P* = 0.0102;  ns |
| 6**d**  CGRP in DRG | n=5 | One-way ANOVA  One-way ANOVA | **Ipsi:**  F(2,12)=441.8, P<0.0001  **Contr:**  F(2,12)=40.31, P<0.0001 | Turkey’ multiple  comparison  Turkey’ multiple  comparison | Saline vs. LF-PENS, *P* <0.0001;  LF-PENS vs. HNK+LF-PENS, *P* <0.0001;  Saline vs. LF-PENS, *P* <0.0001;  LF-PENS vs. HNK+LF-PENS, *P* <0.0001; |
| 6**d**  CGRP in SDH | n=5 | One-way ANOVA  One-way ANOVA | **Ipsi:**  F(2,12)=19.85, P=0.0002  **Contr:**  F(2,12)=8.946, P=0.0042 | Turkey’ multiple  comparison  Turkey’ multiple  comparison | Saline vs. LF-PENS, *P* = 0.0003;  LF-PENS vs. HNK+LF-PENS, *P* = 0.0005;  Saline vs. LF-PENS, *P* = 0.0175;  LF-PENS vs. HNK+LF-PENS, *P* = 0.0049; |
| 6**f**  CGRP in DRG | n=5 | Kruskal-Wallis test  One-way ANOVA | **Ipsi:**  Kruskal-Wallis statistic=9.637, P=0.0018  **Contr:**  F(2,12)=32.65, P<0.0001 | Dunn's multiple comparison  Turkey’ multiple  comparison | Saline vs. LF-PENS, *P* = 0.0111;  LF-PENS vs. LF-PENS +HNK, *P* = 0.0483;  Saline vs. LF-PENS, *P* = 0.0002;  LF-PENS vs. LF-PENS +HNK, *P* <0.0001; |
| 6**f**  CGRP in SDH | n=5 | One-way ANOVA  One-way ANOVA | **Ipsi:**  F(2,12)=14.19, P=0.0007  **Contr:**  F(2,12)=12.32, P=0.0012 | Turkey’ multiple  comparison  Turkey’ multiple  comparison | Saline vs. LF-PENS, *P* = 0.0007;  LF-PENS vs. LF-PENS +HNK, *P* = 0.0067;  Saline vs. LF-PENS, *P* = 0.0013;  LF-PENS vs. LF-PENS +HNK, *P* = 0.0087; |
| 6**i**  3 h Iba1 | n=5 | One-way ANOVA  One-way ANOVA | **Ipsi:**  F(2,12)=11.06, P=0.0019  **Contr:**  F(2,12)=11.31, P=0.0017 | Turkey’ multiple  comparison  Turkey’ multiple  comparison | Saline vs. LF-PENS, *P* = 0.0018;  Saline vs. HNK+LF-PENS, *P* = 0.0163;  Saline vs. LF-PENS, *P* = 0.0017;  Saline vs. LF-PENS +HNK, *P* = 0.0131; |
| 6**i**  30 d Iba1 | n=5 | One-way ANOVA  One-way ANOVA | **Ipsi:**  F(2,12)=18.35, P=0.0002  **Contr:**  F(2,12)=8.310, P=0.0054 | Turkey’ multiple  comparison  Turkey’ multiple  comparison | Saline vs. LF-PENS, *P* = 0.0003;  Saline vs. LF-PENS +HNK, *P* = 0.0016;  Saline vs. LF-PENS, *P* = 0.0109;  Saline vs. LF-PENS +HNK, *P* = 0.0105; |
| 7**a**  Trpa1 | n=6 | One-way ANOVA  One-way ANOVA | **Ipsi:**  F(2,15)=14.16, P=0.0004  **Contr:**  F(2,15)=0.8393, P=0.4513 | Turkey’ multiple  comparison  Turkey’ multiple  comparison | Saline vs. LF-PENS, *P* =0.0229;  LF-PENS vs. HNK+LF-PENS, *P* =0.0002;  ns |
| 7**a**  Trpv1 | n=6 | One-way ANOVA  Kruskal-Wallis test | **Ipsi:**  F(2,15)=13.94, P=0.0004  **Contr:**  Kruskal-Wallis statistic=4.110, P=0.1279 | Turkey’ multiple  comparison  Dunn's multiple comparison | Saline vs. LF-PENS, *P* =0.0381;  LF-PENS vs. HNK+LF-PENS, *P* =0.0003;  ns |
| 7**a**  Vglut2 | n=5,6 | One-way ANOVA  Kruskal-Wallis test | **Ipsi:**  F(2,12)=8.680, P=0.0040  **Contr:**  Kruskal-Wallis statistic=5.402, P=0.0611 | Turkey’ multiple  comparison  Dunn's multiple comparison | Saline vs. LF-PENS, *P* =0.0473;  LF-PENS vs. HNK+LF-PENS, *P* =0.0032;  ns |
| 7**a**  3 h Nr2b in DRG | n=5-7 | One-way ANOVA  One-way ANOVA | **Ipsi:**  F(2,15)=0.9244, P=0.4182  **Contr:**  F(2,15)=1.399, P=0.2773 | Turkey’ multiple  comparison  Turkey’ multiple  comparison | ns  ns |
| 7**a**  1 d Nr2b in SDH | n=6,7 | Kruskal-Wallis test  Kruskal-Wallis test | **Ipsi:**  Kruskal-Wallis statistic=12.18, P=0.0002  **Contr:**  Kruskal-Wallis statistic=4.010, P=0.1355 | Dunn's multiple comparison  Dunn's multiple comparison | Saline vs. LF-PENS, *P* =0.0395;  LF-PENS vs. LF-PENS+HNK, *P* =0.0021;  ns |
| 7**c**  TRPA1 | n=5 | One-way ANOVA  One-way ANOVA | **Ipsi:**  F(2,12)=72.69, P<0.0001  **Contr:**  F(2,12)=9.808, P=0.0030 | Turkey’ multiple  comparison  Turkey’ multiple  comparison | Saline vs. LF-PENS, *P* <0.0001;  LF-PENS vs. HNK+LF-PENS, *P* <0.0001;  Saline vs. LF-PENS, *P* =0.0025;  LF-PENS vs. HNK+LF-PENS, *P* =0.0352; |
| 7**c**  TRPV1 | n=5 | One-way ANOVA  One-way ANOVA | **Ipsi:**  F(2,12)=107.1, P<0.0001  **Contr:**  F(2,12)=100.3, P<0.0001 | Turkey’ multiple  comparison  Turkey’ multiple  comparison | Saline vs. LF-PENS, *P* <0.0001;  Saline vs. HNK+LF-PENS, *P* =0.0125;  LF-PENS vs. HNK+LF-PENS, *P* <0.0001;  Saline vs. LF-PENS, *P* <0.0001;  LF-PENS vs. HNK+LF-PENS, *P* <0.0001; |
| 7**c**  Vglut2 | n=5 | One-way ANOVA  One-way ANOVA | **Ipsi:**  F (2, 12) = 117.4, P<0.0001  **Contr:**  F(2,12)=81.35, P<0.0001 | Turkey’ multiple  comparison  Turkey’ multiple  comparison | Saline vs. LF-PENS, *P* <0.0001;  Saline vs. HNK+LF-PENS, *P* <0.0001;  LF-PENS vs. HNK+LF-PENS, *P* =0.0002;  Saline vs. LF-PENS, *P* <0.0001;  Saline vs. HNK+LF-PENS, *P* <0.0001;  LF-PENS vs. HNK+LF-PENS, *P* = 0.0129; |
| 7**f** | n=5 | One-way ANOVA  One-way ANOVA | **TRPA1 30 d**  **Ipsi:**  **Brown-Forsythe test:**  P= 0.1701  F (2, 12) = 33.37, P<0.0001  **Contr:**  **Brown-Forsythe test:**  P= 0.6534  F (2, 12) = 30.03, P<0.0001 | Turkey’ multiple  comparison  Turkey’ multiple  comparison | Saline vs. LF-PENS, *P* <0.0001;  LF-PENS vs. LF-PENS +HNK, *P* <0.0001;  Saline vs. LF-PENS, *P* <0.0001;  LF-PENS vs. LF-PENS +HNK, P=0.0008 |
| 7**i**  TRPA1 | n=3 | One-way ANOVA  One-way ANOVA  One-way ANOVA  One-way ANOVA | **3 h DRG:**  F(2,6)=14.67, P=0.0049  **3 h SDH:**  F(2,6)=20.25, P=0.0021  **30 d DRG:**  F(2,6)=24.65, P=0.0013  **30 d SDH:**  F(2,6)=15.64, P=0.0042 | Turkey’ multiple  Comparison  Turkey’ multiple  Comparison  Turkey’ multiple  Comparison  Turkey’ multiple  comparison | Saline vs. LF-PENS, *P=*0.0046;  LF-PENS vs. HNK+ LF-PENS, *P=* 0.00225;  Saline vs. LF-PENS, *P=*0.0017;  LF-PENS vs. HNK+ LF-PENS, *P*=0.00308;  Saline vs. LF-PENS, *P=*0.001;  LF-PENS vs. HNK+ LF-PENS, *P*= 0.033;  Saline vs. LF-PENS, *P=*0.092;  LF-PENS vs. HNK+ LF-PENS, *P*=0.0054; |
| 7**i**  TRPV1 | n=3 | One-way ANOVA  One-way ANOVA  One-way ANOVA  One-way ANOVA | **3 h DRG:**  F(2,6)=20.74, P=0.002  **3 h SDH:**  F(2,6)=38.89, P=0.0004  **30 d DRG:**  F(2,6)=12.19, P=0.0077  **30 d SDH:**  F(2,6)=28.81, P=0.0008 | Turkey’ multiple  comparison  Turkey’ multiple  Comparison  Turkey’ multiple  Comparison  Turkey’ multiple  comparison | Saline vs. LF-PENS, P=0.0018;  LF-PENS vs. HNK+ LF-PENS, P= 0.0111;  Saline vs. LF-PENS, P=0.0005;  LF-PENS vs. HNK+ LF-PENS, P=0.0008;  Saline vs. LF-PENS, P=0.0091;  LF-PENS vs. HNK+ LF-PENS, P= 0.0184;  Saline vs. LF-PENS, P=0.0014;  LF-PENS vs. HNK+ LF-PENS, P=0.0015; |
| 8**c** | - | RM  one-way ANOVA | **-** | Bonferroni's multiple  comparison | - |
| 8**f** | - | RM  one-way ANOVA | **-** | Bonferroni's multiple  comparison | - |
| 9**b** | - | RM  one-way ANOVA | **-** | Bonferroni's multiple  comparison | - |
| 9**d**  p-ERK | n=5 | One-way ANOVA | F(3,16)=5.113, P=0.0114 | Turkey’ multiple  comparison | HHBS vs. 30 μM HNK, P= 0.0069; |
| 9**d**  CGRP | n=5 | One-way ANOVA | F(3,16)=2.783, P=0.0747 | Turkey’ multiple  comparison | HHBS vs. 30 μM HNK, P= 0.0432; |
| 9**d**  Merge | n=5 | One-way ANOVA | F(3,16)=5.296, P=0.0100 | Turkey’ multiple  comparison | HHBS vs. 10 μM HNK, P= 0.0478;  Saline vs. 30 μM HNK, P= 0.0075; |
| 10**b**  TRPA1 | n=11 | One-way ANOVA | F(4,50)=27.79, P<0.0001 | Turkey’ multiple  comparison | HHBS vs. Formal, P<0.0001;  Formal vs. HNK+Formal, P<0.0001;  Formal vs. Menthol+Formal, P<0.0001;  Formal vs. HNK&menthol+Formal, P <0.0001; |
| 10**b**  VGLUT2 | n=11 | One-way ANOVA | F(4,50)=27.42, P<0.0001 | Turkey’ multiple  comparison | HHBS vs. Formal, P<0.0001;  Formal vs. HNK+Formal, P<0.0001;  Formal vs. Menthol+Formal, P<0.0001;  Formal vs. HNK&menthol+Formal, P <0.0001; |
| 10**b**  CGRP | n=11 | One-way ANOVA | F(4,50)=10.99, P<0.0001 | Turkey’ multiple  comparison | HHBS vs. Formal, P =0.0002;  Formal vs. HNK+Formal, P= 0.0452;  Formal vs. Menthol+Formal, P<0.0001;  Formal vs. HNK&menthol+Formal, P <0.0001; |
| 10**c**  Trpa1 | n=9 | One-way ANOVA | F(4,40)=4.326, P=0.0053 | Turkey’ multiple  comparison | Formal vs. Menthol+Formal, P= 0.0463;  Formal vs. HNK&menthol+Formal, P = 0.0194; |
| 10**c**  Vglut2 | n=9 | One-way ANOVA | F(4,40)=23.24, P<0.0001 | Turkey’ multiple  comparison | HHBS vs. Formal, P= 0.0048;  Formal vs. HNK+Formal, P<0.0001;  Formal vs. Menthol+Formal, P<0.0001;  Formal vs. HNK&menthol+Formal, P <0.0001; |
| 10**c**  Calca | n=9 | One-way ANOVA | F(4,40)=22.42, P <0.0001 | Turkey’ multiple  comparison | HHBS vs. Formal, P =0.0450;  Formal vs. HNK+Formal, P<0.0001;  Formal vs. Menthol+Formal, P<0.0001;  Formal vs. HNK&menthol+Formal, P =0.0044; |
| 10**c**  Bdnf | n=9 | One-way ANOVA | F(4,40)=21.52,  P <0.0001 | Turkey’ multiple  comparison | Formal vs. HNK+Formal, P =0.0063;  Formal vs. Menthol+Formal, P=0.0001;  Formal vs. HNK&menthol+Formal, P <0.0001; |
| 10**d** | n=5 | Two-way ANOVA | - | Turkey’ multiple  comparison | - |
| 10**e** | n=5 | Two-way ANOVA | - | Turkey’ multiple  comparison | - |
